# Supplementary material for: Genome-Wide Searching Single Nucleotide-Polymorphisms (SNPs) and SNPs-Targeting a Multiplex Primer for Identification of Common Salmonella Serotypes
Source: Pathogens. 2022 Sep 21;11(10):1075. doi: 10.3390/pathogens11101075 (PMC9611365; doi:10.3390/pathogens11101075)
Supplement: Supplementary file 1 [file pathogens-11-01075-s001.zip › Supplementary lists (table and figure).pdf]

Supplementary Tables legend

Tables S1. A. Information of single nucleotide polymorphism (SNPs) sites on the alignment of the reference and comparing *Salmonella bogori* strains. B. The position of single nucleotide polymorphism (SNPs) sites on the alignment of reference and comparing *Salmonella enterica* subspecies enterica serovar strains.

Table S2. Information of success rate with SNP-encompassing primers amplification.

Table S3. Information of target six *Salmonella enetrica* subspecies enterica serovars, their amplified sequences with encompassing primers, alignments of amplified sequences, and searching SNPs on the alignment, and design SNP-based primers on the respective align genes.

Table S4. List of *Salmonella* serovar-specific single nucleotide polymorphisms (SNP)-based primers with respective genes used in this study.

Tables S5. A-B. A. Specificity test of newly designed SNP-based primers with reference six serovars. B. Final selection of *Salmonella* specific SNP-based primers

Supplementary Figs legend:

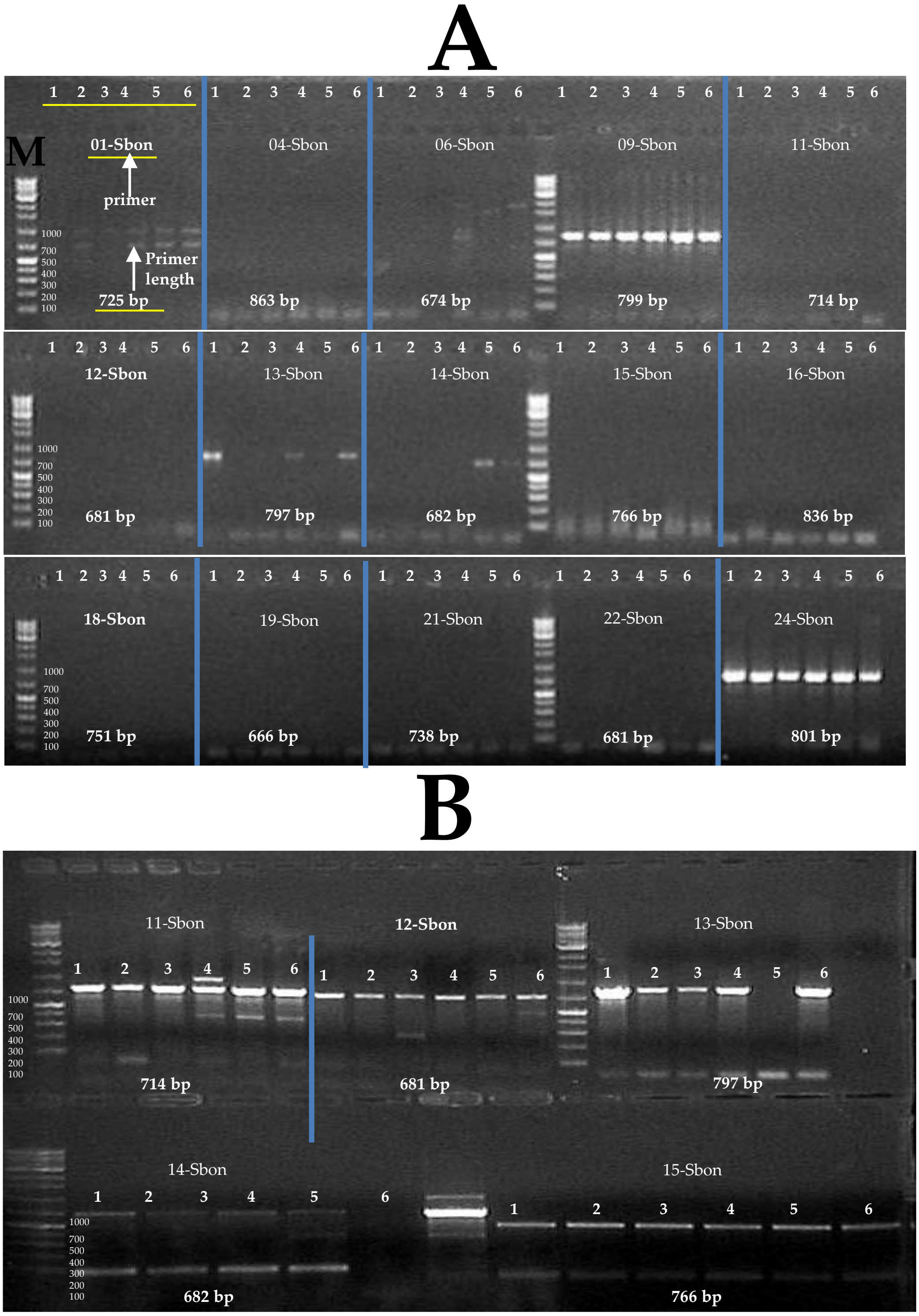

Figure S1. Desire single nucleotide polymorphisms (SNPs) encompassing PCR amplification of six target *Salmonella* with 15 primer sets. PCR band 'M' indicates DNA 100 bp marker. The gel lane numbers are provided in each section (1-6): lane No.1= *Salmonella enterica* subspecies *enterica* serovar Typhimurium (NCCP-14760); No.2= *S. e.* Enteritidis (NCCP-14545); No.3= *S. e.* Agona (NCCP-12231); No.4= *S. e.* enterica (NCCP-15756); No.5= *S. e.* Typhi (NCCP-14641); No.6= *S. e.* Abony. **A.** First PCR amplifies all 15 primer sets but fails to produce all primers with target band. **B.** Second PCR amplification with primers of 11-, 12-, 13-, 14- and 15-Sbon primer sets which did not produce the target band during the first PCR. The three (4-, 6, and 22-Sbon) primer sets could not produce any band during both PCR cycles. SNP-based target band length was indicated by a yellow color underline. Detailed sequence information is provided in table S3

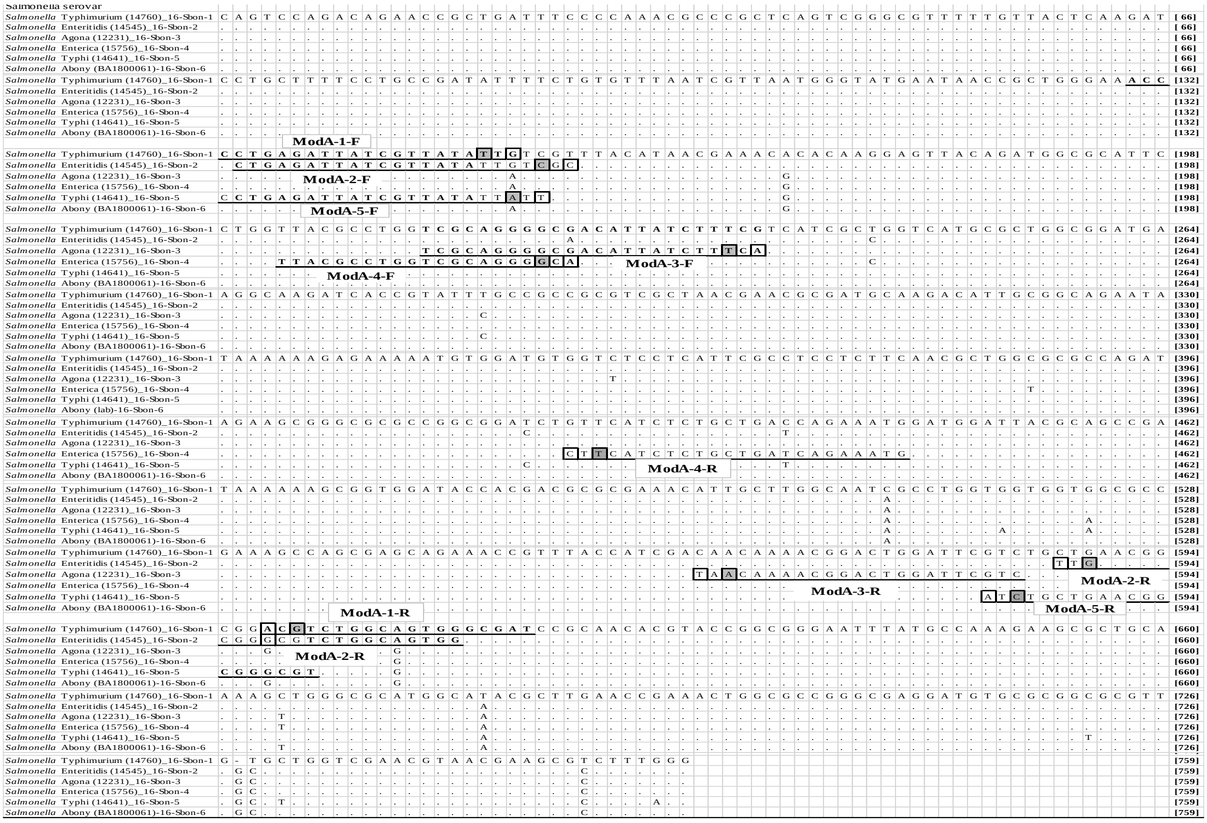

Figure S2. The alignment of "16-Sbon" primer amplified gene sequences of (Molybdate-binding periplasmic protein, *modA*) of target six *Salmonella* serotypes. Primer pair is indicated by underline and italicized region, natural SNP base is marked by a square shape, and artificial mutated SNP base is marked by a black shaded square shape. Here, the SNP-based serotype-specific *Salmonella* detective five primer pairs were presented and designed. The number of sites was 759; "-" indicates indel; "?" indicates missing bases; ".dot" indicates identical bases
