# Supplementary material for: Genome-Wide Searching Single Nucleotide-Polymorphisms (SNPs) and SNPs-Targeting a Multiplex Primer for Identification of Common Salmonella Serotypes
Source: Pathogens. 2022 Sep 21;11(10):1075. doi: 10.3390/pathogens11101075 (PMC9611365; doi:10.3390/pathogens11101075)
Supplement: Supplementary file 1 [file pathogens-11-01075-s001.zip › Table S3.pdf]

Table S3. The information of target six *Salmonella enetrica* subspecies enterica serovars, their amplified sequences with encompassing primers, alignments of amplified sequences, and searching SNPs on the alignment, and design SNP-based primers on the respective align genes.

No	*Salmonella* serovars	Catalog No.	Source strains
1	*Salmonella enterica* subspecies enterica serovar Typhimurium (*Salmonella* Typhimurium)	NCCP-14760	National Culture Collection of Pathogens
2	*Salmonella* Enteritidis	NCCP-14545	National Culture Collection of Pathogens
3	*Salmonella* Agona	NCCP-12231	National Culture Collection of Pathogens
4	*Salmonella enterica*	NCCP-15756	National Culture Collection of Pathogens
5	*Salmonella Typhi*	NCCP-14641	National Culture Collection of Pathogens
6	*Salmonella* Abony	BA1800061	Veterinary Culture Collection Center

The PCR amplification of 1st time PCR with the primer sets (01-, 09-, 13-, 14-, 24-Sbon) are as following:

The amplified sequences of six *Salmonella* serovars (the provided serial no. was presented in the above table: 1-6; SNP-encompassing primers, gene name) are presented below: **for example, primer name (01-Sbon-2); primer serial-short form of *Salmonella bongori*-*Salmonella* serovar serial no. (1-6)**

01. Sbon F/R, Gene Name: Conserved hypothetical protein (*SBG*) SBG_RS00105

**01-Sbon-F:**	**GGGGAAATGTTGGCGGGA**
**01-Sbon-R:**	**TTATGCCCGGTGCCATGG**

>01-Sbon-2 **(name of primer-serial no. - short form of *Salmonella bongori* -*Salmonella* serovar serial no in the above table)**

CCCACCGTCTCAAGAACTGATTGTCTGTAGTACATCAGCAGGAGACCCTGTTACATCAACTGCTGGAGGGCGACCTGCGA

CAGCGGGTAATGGACGCGATTGTAATCGCTGCCGGAACGCGAGCAACTGGTGTTAACGCTGTATTACCAGGAAGAGCTCA

ACCTCAAAGAGATTGGCGCGGTACTGGAAGTTGGCGAATCGCGGGTCAGCCAGTTGCATAGTCAGGCCATCAAACGATTA

CGCACCAAACTGGGTAAGTTATAGGTCGCGCATGGTCGCACCCGAAAAGTGCCGCACAACGTATAGACTACCAGGAGTTC

TCATGACGGTGCAGCAACCTAAAAGGCGGCCCTTGAGCCGCTATCTTAAAGACTTTAAACACAGCCAGACGCATTGCGCG

CATTGTCACAAACTGCTCGACCGCATTACGCTGGTTCGCCGTGGCAAGATCGTTAATAAAATCGCTATTTCACAGCTGGA

TATGCTACTTGACGACGCTGCCTGGCAGCGGGAGCAGAAGGAGTGGGTGGCGCTGTGTCGCTTTTGCGGCGATTTGCACT

GCAAAAAGCAGAGTGATTTTTTCGATATTATCGGTTTCAAGCAGTATTTGTTTGAACAAACCGAGATGAGCCATGGCACC

GGGGCATAAA

>01-Sbon-4

AAACCGCAAATCGAAACTGAGTGTATGAGACATCAACAGGAGAACCCGTTACATCAACTGCTGGAGGGCGACCTGCGACA

GCGGGTAATGGATGCGATTGAATCGCTGCCGGAACGCGAGCAACTGGTGTTAACGCTGTATTACCAGGAAGAGCTCAATC

TCAAAGAGATTGGCGCGGTACTGGAAGTCGGCGAATCGCGGGTCAGCCAGTTGCATAGTCAGGCCATCAAACGATTACGC

ACCAAACTGGGTAAGTTATAGGTCGCGCATGATCGCACCCGAAAAGTGCCGCACAACGTATAGACTACCAGGAGTTCTCA

TGACGGTGCAGCAACCTAAAAGGCGGCCTTTGAGCCGCTATCTTAAAGACTTTAAACACAGCCAGACGCATTGCGCGCAT

TGTCACAAACTGCTCGACCGCATTACGCTGGTTCGCCGTGGCAAGATCGTTAATAAAATCGCTATTTCACAGCTGGATAT

GCTACTTGACGACGCTGCCTGGCAGCGGGAGCAGAAGGAGTGGGTGGCGCTGTGTCGCTTTTGCGGCGATTTGCACTGCA

AAAAGCAGAGTGATTTTTTCGATATTATCGGTTTCAAGCAGTATTTGTTTGAACAAACCGAGATGAGCCATGGCACCGGG

GCATAAA

>01-Sbon-5

TGTAATACCCGACTGGTGTCTGAGACATCAGCAGGAAACCCGTTACATCAACTGCTGGAGGGCGACCTGCGACAGCGGGT

AATGGATGCGATTGAATCGCTGCCGGAACGCGAGCAACTGGTGTTAACGCTGTATTACCAGGAAGAGCTCAATCTCAAAG

AGATTGGCGCGGTACTGGAAGTCGGCGAATCGCGGGTCAGCCAGTTGCATAGTCAGGCCATCAAACGATTACGCACCAAA

CTGGGTAAGTTATAGGTAGCGCATGATCGCACCCGAAAAGTGCCGCACAACATATAGACTACCAGGAGTTCTCATGACGG

TGCAGCAACCTAAAAGGCGGCCCTTGAGCCGCTATCTTAAAGACTTTAAACACAGCCAGACGCATTGCGCGCATTGTCAC

AAATTGCTCGACCGCATTACGCTGGTTCGCCGTGGCAAGATCGTTAATAAAATCGCTATTTCACAGCTGGATATGCTACT

TGACGACGCTGCCTGGCAGCGGGAGCAGAAGGAGTGGGTGGCGTTGTGTCGCTTTTGCGGCGATTTGCACTGCAAAAAGC

AGAGTGATTTTTTCGATATTATCGGTTTCAAGCAGTATTTGTTTGAACAAACCGAGATGAGCCATGGCACCGGGGCATAA

A

>01-Sbon-6

ACTGTCTTCCTCGAACTTGGTGATGAGACATCAGCAGGAAACCCGTTACATCAACTGCTGGAGGGCGACCTGCGACAGCG

GGTAATGGATGCGATTGAATCGCTGCCGGAACGCGAGCAACTGGTGTTAACGCTGTATTACCAGGAAGAGCTCAATCTCA

AAGAGATTGGCGCGGTACTGGAAGTCGGCGAATCGCGGGTCAGCCAGTTGCATAGTCAGGCCATCAAACGATTACGCACC

AAACTGGGTAAGTTATAGGTAGCGCATGATCGCACCCGAAAAGTGCCGCACAACGTATAGACTACCAGGAGTTCTCATGA

CGGTGCAGCAACCTAAAAGGCGGCCCTTGAGCCGCTATCTTAAAGACTTTAAACACAGCCAGACGCATTGCGCGCATTGT

CACAAACTGCTCGACCGCATTACGCTGGTTCGCCGTGGCAAGATCGTTAATAAAATCGCTATTTCACAGCTGGATATGCT

ACTTGACGACGCAGCCTGGCAGCGGGAGCAGAAGGAGTGGGTGGCGCTGTGTCGCTTTTGCGGCGATTTGCACTGCAAAA

AGCAGAGTGATTTTTTCGATATTATCGGTTTCAAGCAGTATTTGTTTGAACAAACCGAGATGAGCCATGGCACCGGGGGC

ATAAA

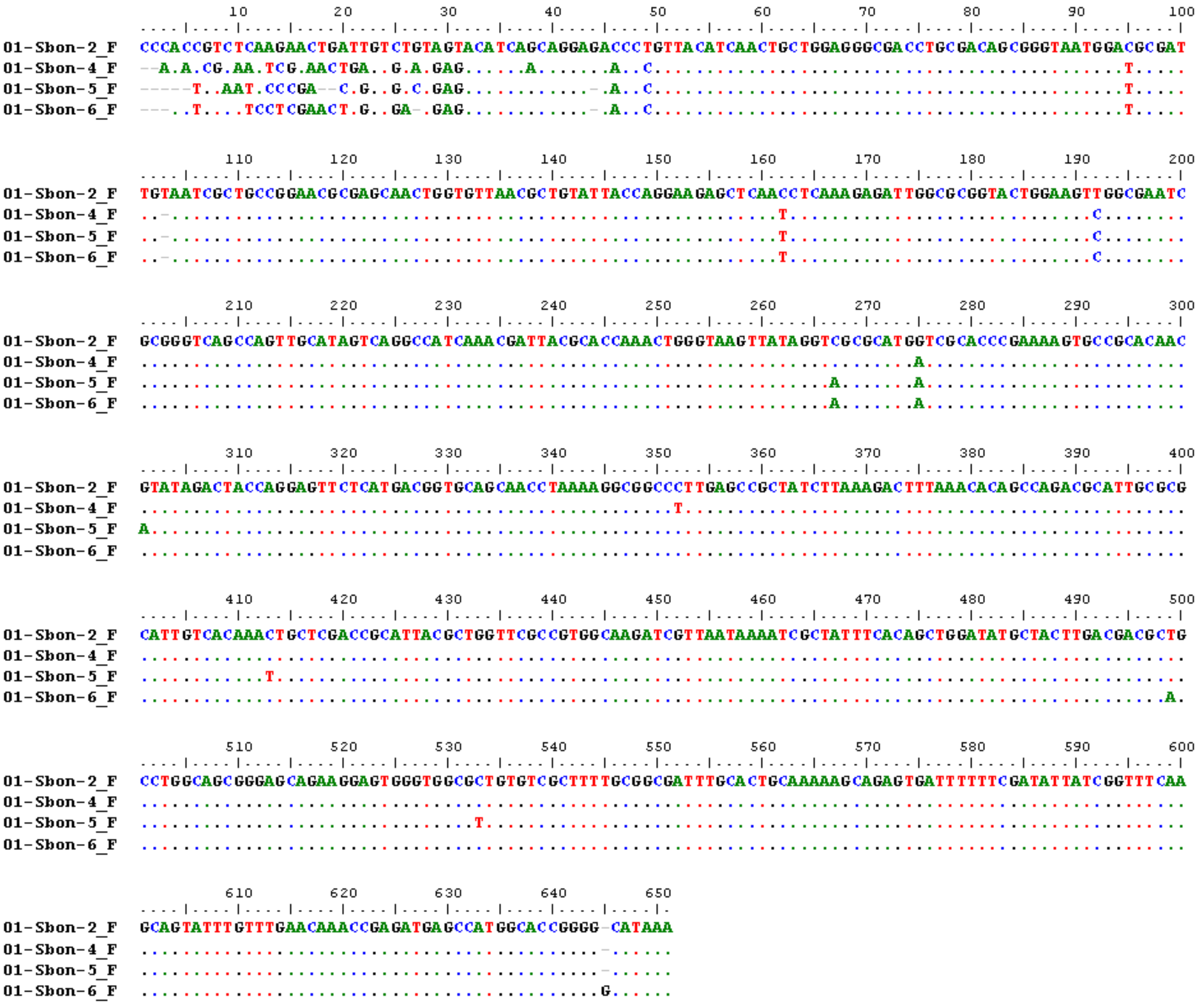

The SNP-based primers were designed based on the above align gene (*SBG*) sequences (natural SNP is marked by red color and artificial mutated bases are marked by yellow color)

SBG-2 F: TTACCAGGAAGAGCTCGAC

SBG-2 R: CGG TGC CAT GGC TCA TCT CG

SBG-2(2)F: GTA AGT TAT AGG TCG CGC ACG G

SBG-2 R: CGG TGC CAT GGC TCA TCT CG

SBG-4 F: GTA AGT TAT AGG TCG CGC AGG A

SBG-4 R: CGG TGC CAT GGC TCA TCT CG

09-Sbon, Gene Name: Penicillin Binding protein gene (*mrcB*)

**09-Sbon-F**	**GGCGTTGAAGAAGCAGCG**
**09-Sbon-R**	**ACGGCCTACCCAGGTGAT**

>09-Sbon-1

CCTTGAACTGCACGCGAGGTGGTAGTGGACGCTTTAGCGGCGAGGTGCGCGCGATGGTTGGCGGGGCGGAGCCGCAGTAT

GCCGGCTATAACCGTGCCATGCAGGCGCGCCGTTCCATCGGGTCGCTGGCGAAACCGGCGACCTATCTGACCGCGTTAAG

TCAGCCGAACTTATACCGTCTGAACACCTGGATTGCCGATGCGCCGATTTCTCTGCGCCAGCCGAATGGTCAGGTCTGGT

CGCCGCAGAACGACGATCGTCGCTACAGCGAAAGCGGGAAAGTGATGCTGGTGGATGCGTTAACTCGCTCAATGAACGTA

CCGACGGTCAATCTGGGGATGGCGTTAGGTCTACCGGCAGTAACCGATACCTGGACGAAGCTCGGCGTGCCGAAAGATCA

GCTCAATCCGGTTCCGGCGATGCTGTTAGGGGCGCTGAACCTGACGCCGATCGAAGTGGCGCAGGCGTTCCAGACTATCG

CCAGCGGCGGAAATCGCGCGCCGTTATCAGCGCTGCGTTCGGTTATTGCGGAAGATGGTAAAGTGCTGTACCAAAGTTAT

CCGCAAGCTGAACGCGCTGTACCGGCACAGGCGGCTTACCTGACGCTCTGGACAATGCAGCAGGTCGTCCAGCGTGGTAC

GGGGCGTCAGCTTGGCGCGAAATATCCGGGTCTGCATCTGGCCGGTAAAACCGGGACGACAAACAACAATGTCGATACCT

GGTTTGCCGGTATCGACGGCAGCCAGGTGACTATCACCTGGGTAGGCCGTAGAT

>09-Sbon-2

GCTGAAATGGCACGCGAGGTGGTGGTGGACGCTTTAGCGGCGAGGTGCGCGCGATGGTTGGCGGGGCGGAGCCGCAGTAT

GCCGGCTATAACCGTGCCATGCAGGCGCGCCGTTCCATCGGGTCGCTGGCGAAACCGGCGACCTATCTGACCGCGTTAAG

TCAGCCGAACTTATACCGTCTGAACACCTGGATTGCCGATGCGCCGATTTCTCTGCGCCAGCCGAATGGTCAGGTCTGGT

CGCCGCAGAACGACGATCGTCGCTACAGCGAAAGCGGGAAAGTGATGCTGGTGGATGCGTTAACTCGCTCAATGAACGTA

CCGACGGTCAATCTGGGGATGGCGTTAGGTTTACCGGCGGTAACCGATACCTGGACGAAGCTCGGCGTGCCGAAAGATCA

GCTCAATCCGGTTCCGGCTATGCTGTTAGGGGCGCTGAACCTGACGCCGATCGAAGTGGCGCAGGCGTTCCAGACTATCG

CCAGCGGCGGAAATCGCGCGCCGTTATCAGCGCTGCGTTCAGTTATTGCGGAAGATGGTAAAGTGCTGTACCAAAGTTAT

CCGCAAGCTGAACGCGCTGTACCGGCACAGGCGGCTTACCTGACGCTCTGGACAATGCAGCAGGTCGTCCAGCGTGGTAC

GGGGCGTCAGCTTGGCGCGAAATATCCGGGTCTGCATCTGGCGGGTAAAACCGGGACGACAAACAACAATGTCGATACCT

GGTTTGCCGGTATCGACGGCAGCCAGGTGACTATCACCTGGGGTAGGCCGTA

>09-Sbon-3

GCGTGACCTGGACGCGAGGTGGTAGTGGACGCTTTAGCGGCGAGGTGCGCGCGATGGTTGGCGGGGCGGAGCCGCAGTAT

GCCGGCTATAACCGTGCCATGCAGGCGCGCCGTTCCATCGGGTCGCTGGCGAAACCGGCGACCTATCTGACCGCGTTAAG

TCAGCCGAACTTATACCGTCTGAACACCTGGATTGCCGATGCGCCGATTTCGCTGCGCCAGCCGAATGGTCAGGTCTGGT

CGCCGCAGAACGACGATCGTCGCTACAGCGAAAGCGGGAAAGTGATGCTGGTGGATGCGTTAACTCGCTCAATGAACGTA

CCsGACGGTCAATCTGGGGATGGCGTTAGGTTTACCGGCGGTAACCGATACCTGGACGAAGCTCGGCGTGCCGAAAGATCA

GCTCAATCCGGTTCCGGCTATGCTGTTAGGGGCGCTGAACCTGACGCCGATCGAAGTGGCGCAAGCGTTCCAGACTATCG

CCAGCGGCGGAAATCGCGCGCCGTTATCAGCGCTGCGTTCGGTTATTGCGGAAGATGGTAAGGTGCTGTACCAAAGTTAT

CCGCAAGCTGAACGCGCCGTACCGGCACAGGCGGCTTACCTGACGCTCTGGACAATGCAGCAGGTCGTCCAGCGTGGTAC

GGGGCGTCAGCTTGGCGCGAAATATCCGGGTCTGCATCTGGCGGGTAAAACCGGGACGACAAACAACAATGTCGATACCT

GGTTTGCCGGTATCGACGGCAGCCAGGTGACTATCACCTGGGTAGGCCGTAGA

>09-Sbon-4

CCTCCACTGGTACGCGAGGTGGTGGTGGACGCTTTAGCGGCGAGGTGCGCGCGATGGTTGGCGGGGCGGAGCCGCAGTAT

GCCGGCTATAACCGTGCCATGCAGGCGCGCCGTTCCATCGGGTCGCTGGCGAAACCGGCGACCTATCTGACCGCGTTAAG

TCAGCCGAACTTATACCGTCTGAACACCTGGATTGCCGATGCGCCGATTTCTCTGCGCCAGCCGAATGGTCAGGTCTGGT

CGCCGCAGAACGACGATCGTCGCTACAGCGAAAGCGGGAAAGTGATGCTGGTGGATGCGTTAACTCGCTCAATGAACGTA

CCGACGGTCAATCTGGGGATGGCGTTAGGTTTACCGGCGGTAACCGATACCTGGACGAAGCTCGGCGTGCCGAAAGATCA

GCTCAATCCGGTTCCGGCTATGCTGTTAGGAGCGCTGAACCTGACGCCGATCGAAGTGGCGCAGGCGTTCCAGACTATCG

CCAGCGGCGGAAATCGCGCGCCGTTATCAGCGCTGCGTTCAGTTATTGCGGAAGATGGTAAAGTGCTGTACCAAAGTTAT

CCGCAAGCTGAACGCGCCGTACCGGCACAGGCGGCTTACCTGACGCTCTGGACAATGCAGCAGGTCGTCCAGCGTGGTAC

GGGGCGTCAGCTTGGCGCGAAATATCCGGGTCTGCATCTGGCCGGTAAAACCGGGACGACAAACAACAATGTCGATACCT

GGTTTTGCCGGTATCGACGGCAGCCAGGTGACTATCACTCTGGGGTAGGCCGTAG

>09-Sbon-5

GCTTCACTGCACTGCGAGGTGGTAGTGGACGCTTTAGCGGCGAGGTACGCGCGATGGTTGGCGGGGCGGAGCCGCAGTAT

GCTGGCTATAACCGTGCCATGCAGGCACGCCGTTCCATCGGGTCGCTGGCGAAACCGGCGACCTATCTGACCGCGTTAAG

TCAGCCGAACTTATACCGTCTGAACACCTGGATTGCCGATGCGCCGATTTCTCTGCGCCAGCCGAATGGTCAGGTCTGGT

CGCCGGAGAACGACGATCGTCGCTACAGCGAAAGCGGGAAAGTGATGCTGGTGGATGCGTTAACTCGTTCAATGAACGTA

CCGACGGTCAATCTGGGGATGGCGTTAGGTTTACCGGCGGTAACCGATACCTGGACGAAGCTCGGCGTGCCGAAAGATCA

GCTCAATCCGGTTCCGGCGATGCTGTTAGGGGCGCTGAACCTGACGCCGATCGAAGTGGCGCAAGCGTTCCAGACTATCG

CCAGCGGCGGAAATCGCGCGCCGTTATCAGCGCTGCGTTCGGTTATTGCGGAAGATGGTAAAGTGCTGTACCAAAGTTAT

CCGCAAGCTGAACGCGCCGTACCGGCACAGGCGGCTTACCTGACGCTCTGGACAATGCAGCAGGTCGTCCAGCGTGGTAC

GGGGCGTCAGCTTGGCGCGAAATATCCGGGTCTGCATCTGGTGGGTAAAACCGGGACGACAAACAACAATGTCGATACCT

GGTTTGCCGGTATCGACGGCAGCCAGGTGACTATCACCTGGGTTAGGCCGTA

>09-Sbon-6

GCTGCACTGACTGCGAGGTGGTAGTGGACGCTTTAGCGGCGAGGTACGCGCGATGGTTGGCGGGGCGGAGCCGCAGTATG

CCGGCTATAACCGTGCCATGCAGGCGCGCCGTTCCATCGGGTCGCTGGCGAAACCGGCGACCTATCTGACCGCGTTAAGT

CAGCCGAACTTATACCGTCTGAACACCTGGATTGCCGATGCGCCGATTTCTCTGCGCCAGCCGAATGGTCAGGTCTGGTC

GCCGCAGAACGACGATCGTCGCTACAGCGAAAGCGGGAAAGTGATGCTGGTGGATGCGTTAACTCGCTCAATGAACGTAC

CGACGGTCAATCTGGGGATGGCGTTAGGTTTACCGGCGGTAACCGATACCTGGACGAAGCTCGGCGTGCCGAAAGATCAG

CTCAATCCGGTTCCGGCGATGCTGTTAGGGGCTCTGAACCTGACGCCGATCGAAGTGGCGCAAGCGTTCCAGACTATCGC

CAGCGGCGGAAATCGCGCTCCGTTATCAGCGCTGCGTTCGGTTATTGCGGAAGATGGTAAAGTGCTGTACCAAAGTTATC

CGCAAGCTGAACGCGCCCGTACCGGCACAGGCGGCTTACCTGACGCTCTGGACAATGCAGCAGGTCGTCCAGCGTGGTACG

GGGCGTCAGCTTGGCGCGAAATATCCGGGTCTGCATCTGGCGGGTAAAACCGGGACGACAAACAACAATGTCGATACCTG

GTTTGCCGGTATCGACGGCAGCCAGGTGACTATCACCTGGGTTAGGCCGTAG

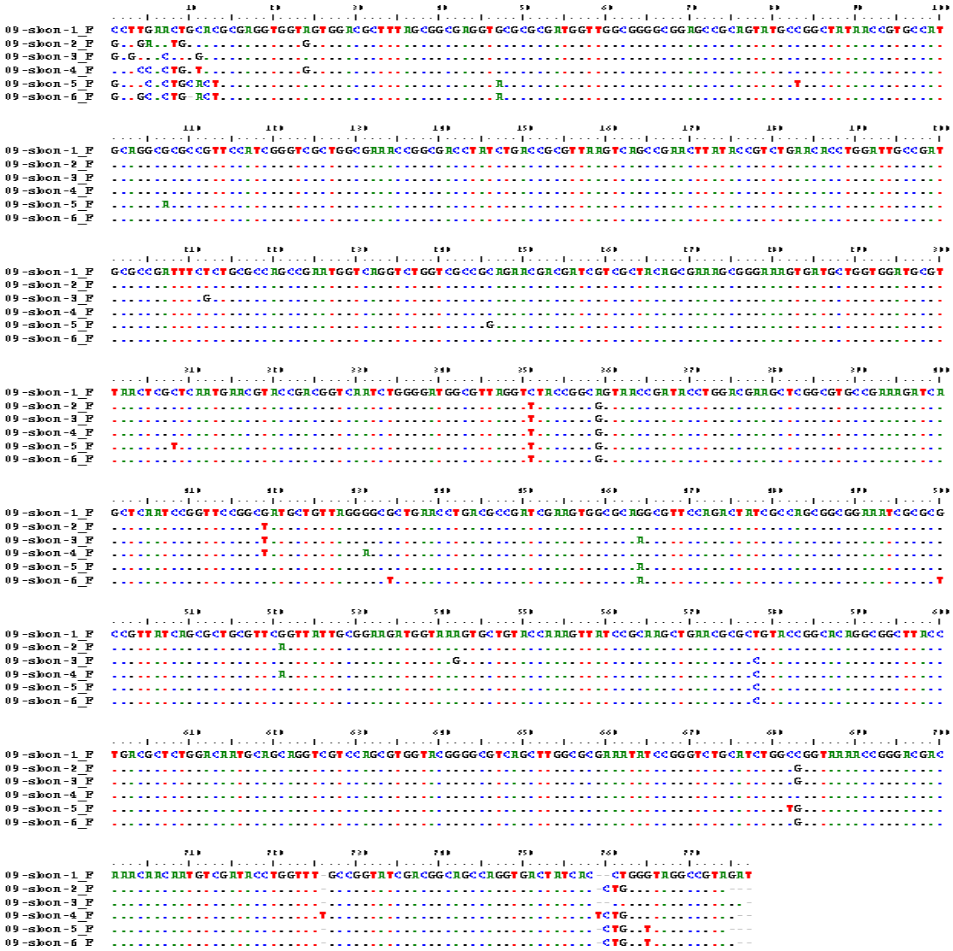

The SNP-based primers were designed based on the above align gene (*mrcB*) sequences (natural SNP is marked by red color and artificial mutated bases are marked by yellow shaded)

mrcB-1-F: TGGCGTTAGGTCTACCGTCA

mrcB-1-R: TTG TCG TCC CGG TTT TAT CG

mrcB-3-F : TTG CCG ATG CGC CGA TTG CG

mrcB-3-R : GGA TAA CTT TGG TAC AGC CCC

mrcB-4-F : GTT CCG GCT ATG CTG TTA AGA

mrcB-4-R :TTG TCG TCC CGG TTT TAT CG

mrcB-5-F : GGC GGA GCC GCA GTA TAC T

mrcB-5-R : TGT CGT CCC GGT TTT ACT CA

mrcB-6-F : ACGCTTTAGCGGCGAGATA

mrcB-6-R : TGT CGT CCC GGT TTT ACT CA

13-Sbon, Gene Name: Pyrimidine-specific ribonucleoside hydrolase *(rihB)*

**013-Sbon-F:**	**GCTGTACCTGCCGACTGG**
**13-Sbon-R:**	**GTTCCCCACGGGCTATGG**

>13-Sbon-1

CAAAGCTTTTTTTAGTTTCTTGCAAGTAAATGCAATTGCAGCATTAACGAAGAAGGAAGTGATCTGGTTAACACTGTAAT

GTAAAAAATGGGTAACGATACGGTTTTATCATGTTGTTGTGTTGTTAATTACGGTAAAGGTGTTATAGAAACAAAATGTA

ACATCTCTCTGGAACACCCAAACGGACAACAACTATGAATAAATCAGGGAAATACCTCGTCTGGACAGCGCTCTCAGTAT

TGGGTGCGTTTGCCCTGGGCTATATTGCGTTAAATCGTGGGGAACAGATCAACGCGCTATGGATCGCACTGGGCGTCGGT

CTGTGTCTATCTTATTGCGTATCGTTTTTATGGGCTCTATATCGCCAAAAAAGTGCTGGCGGTTGACCCAACGCGTATGA

CGCCCGCGGTACGTCATAATGATGGTCTGGATTATGTCCCGACCGATAAGAAAGTGCTTTTTGGTCACCATTTTGCGGCC

ATTGCTGGCGCAGGTCCGCTGGCCTGGCCGGTACTGGCGGCGCAGATGGGCTATCTGCCGGGGATGATCTGTTTGCT

>13-Sbon-4

TTCACACCATAGTATTCTGGCAATAAATTGCATTGCAGCATTAACGAAGAAAGGAAGTGTTCTGGTTAGACTGTAATGAA

AAATGGGAAATTTTCGAATAATTTGTGTGGTGTTAATTACGGTAAGGCTCCTTGAAACAAAATGAACATCTCTCTGGAAC

ACCCAAACGTACAACTACTATGAATAAATCACGCAAAT

>13-Sbon-6

CACCGGGTAGGTAATTCTGGCAAGTAAATGCATTGCAGCATTAACGAAGAAAGGAAGTGATCTGGTTAACACTGTAATGT

AAAAAATGGGTAAAATTCGGATGTATCATGTTGTTGTGTTGTTAATTACGGTAAAGGTGTTATAGAAACAAAATGTAACA

TCTCTCTGGAACACCCAAACGGACAACAGCTATGAATAAATCAGGGAAATACCTCGTCTGGACAGCGCTCTCAGTATTGG

GTGCGTTTGCCCTGGGCTATATTGCGTTAAATCGTGGGGAACAGATCAACGCGCTATGGATCGTGGTGTCTTTTTTTCTG

TGTTTATCTTATTGCGTATCGTTTTTATGGGCTCTATATCGCCAATAAAGTGCTGGCGGTTGACCCTACGCGTATTA

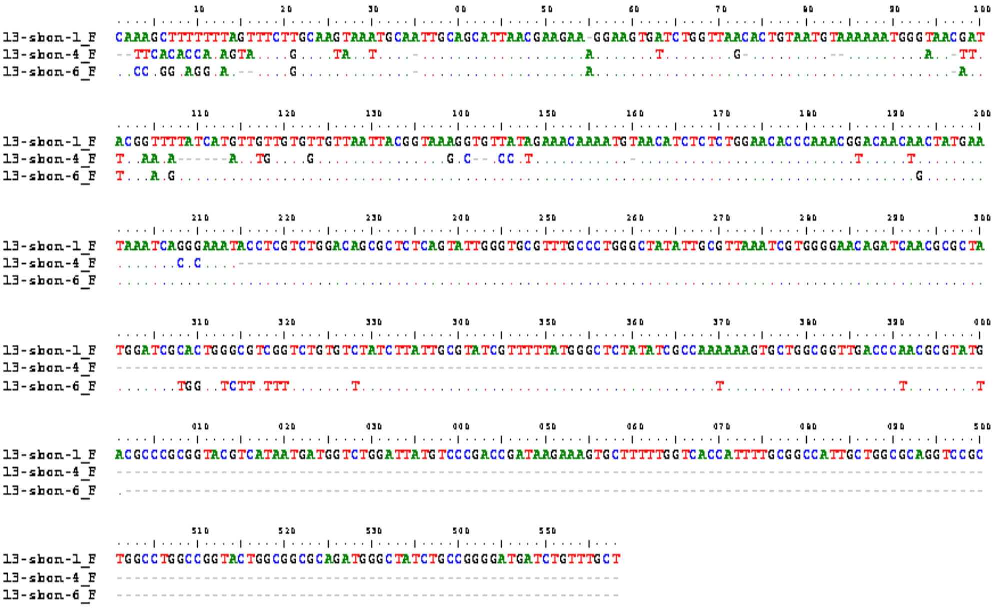

The above align gene (rihB)did not produce the good quality gene sequences of all target Salmonella, where we did not find any suitable SNPs. Second PCR was conducted, and the sequence was showed it latter

14-Sbon, Gene Name: Pyrimidine-specific ribonucleoside hydrolase(*rihA*)

**14-Sbon-F**	**TCCCCTGTGTTTCGACGC**
**14-Sbon-R**	**ACGCCGGATAAGACGCTG**

>14-sbon-5

TGGGCTTGCCTTGGTTGAAATCTCAGGGTTTCAGCAGCCAGGCGATAGTGCAAGGGTCGTGCAGCGGCGCGCCGACAAAT

CCCCATTTTTCGTCTTTGTGGTATTCAAAGAAGAAGTCGAGTAGTTCAGCCACGATCGTCGAAATTGGATTGCCGATGTC

GCGGAAACGCTCGATATCGGCGGCGTGAATCTGCGCCTTATGGGTGACGTCCAGCCCGGCCATCACCACCGGAATGCCTG

ACTGGAAGACAATTTCTGCCGCTTCCGGATCGACGTAAATATTAAATTCCGCAGCGGGCGTCCAGTTACCCAGCGCCATC

GCGCCGCCCATGATCACAATGCGGGCGATTTTCGTATGCAGTTCCGGATGGCTATTCAGCAGCAGCGCCACGTTGGTCTG

TGGGCCAGTGGAGACGATGGTGACCGGCTGTGCGCTCTCGCGCAGTGTTTTTGCCATCAGCTCAACGGCAGTGCCGCTTT

GCGGGGCGAATGACGGCTCGGGCAGCGCCGGGCCGTCAAGACCGCTTTCGCCGTGAACGTTATCAGCGATAATCAGTTCG

CGCATGAGCGGTTTCACCGCGCCGCCCGCCACAGGAATATCCGGGCGTTTGAGCAGTGTCATCATCCCGCAGCACGTT

>14-sbon-6

TTCTTCCGATAGGCAGCTTGTAATCTCAAAGTTCAGCAGCCAGGCGATAGTGCAAGGGTCGTGCAGCGGCGCGCCGACAA

ATCCCCATTTTTCGTCTTTGTGGACAATATAAAGAATCCAGCAGCTCGGGGACGATGGTGGAAATGTTTTTGCCAATGGC

GCGAAAGCGTTCGATGTCGGCGGCGTGAATCTTCTCCTTATGGATGACATCCCCCCCGGCCATCACCAACGGAATGCCTG

ACTGGAAGACGATTTTCCCCTCTTTTGGTTTGATTGAATTTTTAACTCCGCCGCGATCGTCCTTTTGTTTATCTTCATTT

TTTCCGCATTTTTTATTTTCTTTTATTTTTTTTTCTTTTCTATTTTCTATTTTTTTGTATTCCTTTG

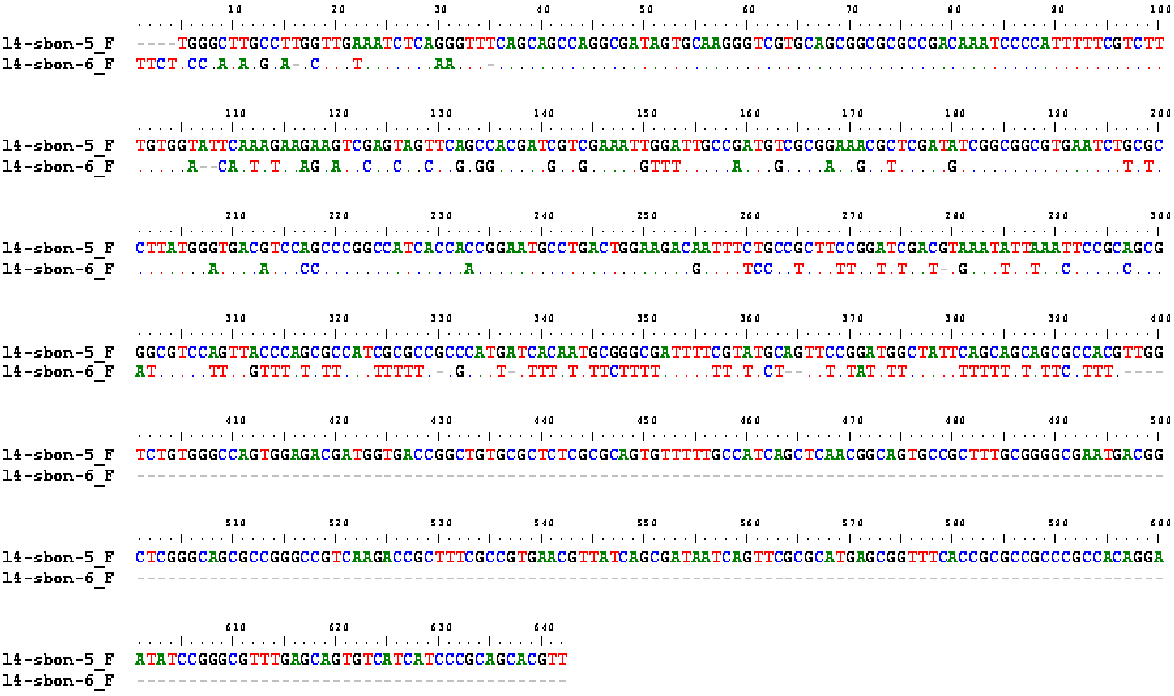

The above alignment of gene, Pyrimidine-specific ribonucleoside hydrolase (rihA) did not produce the all target sequences of Salmonella, where we did not find any suitable SNPs.

<u>24-Sbon, Gene Name: Candidate type three secretion system effector protein (*sopA*)</u>

**24-Sbon-F**	**GTAGTGTGGCGGGCTGAG**
**24-Sbon-R**	**CTGGTAAGCGTGCTGGCC**

>24-sbon-1

TGAGGTAGGGTCAAAATGGTGGCAGACGCGACACTGAGGCATCATCAATCAACTTCACGGTGGAATGTTTCTGGTTCGTG

GCGGCAATAAACTGCTGGTTCAGCTCCCCGTGCTCTTCAGTGCTGAGGATGATATCAATCAGCTCCCCAAAGCGTCGCTG

CGTGGCCTGCTGATTAAATGCGGCGAGAAACACCGGAAAGTCATGACGGAATAACTCTACCGGAGAGATTTGCGCGGTGG

AAACGTTCTCTCCGGCTCGCAGCAGGTAAAAGTTGTCCCATGCAGTGTCAGGAGTAGGGTTTAGCATTGTTAACAGCGTG

TCAGTGGAGAGCATCATCGCCGTGTCTGAATCCTGGGAGGAAAGCAGCAGGAAATTATCTGCAGCGCGGGTTGTCCAGTC

CGGGATGCCGTCATAATTGCCGAAGAGCCCATTATGGATGTGGGGAGACACCGCCGGGTGAGCAAGATACTGTTCATAAA

GCGCTGCAGCTTTCTGGCGGGTGCCATCAGTGCAGTCCACGCCTGTCATACAGTGTAAAATCACCTGATTAAAGAGTCCG

CTGTGAGTGGTCATGGCGGCGCTGTTCCTGTCAAACGAATCCAGCAACACAGGCAGTATGCGCTGCTGATAATATGTGTC

AGGAGTCGGCAGGGGAACGTCATTATAGGTCTGGCACTGGTGGGCAAAAAAACGTTCCTGAACAGGCCCGAGCCACTCGC

GGATAAGCGCACTTTTGCGTATAGGGGAGGGTGGGCCAGCACGCCTCACTCAGACAAATTACTATTTCGCCTGTTGTCCC

TTGTTCGATTCCTGGCCTCTTATTATCTGTCTGGTATATCGTCTTTTGTTCTTTTTTTCTTTCTTTATAGGCATAATTCC

TCCCCCCCTCACCTGACAATGTTTTT

>24-sbon-2

GACGGTAGGAGCAATGGTGGCAGACGCGACACTGAGGCATCATCAATCAACTTCACGGTGGAATGTTTCTGGTTCGTGGC

GGCAATAAACTGCTGGTTCAGCTCCCCGTGCTCTTCAGTGCTGAGGATGATATCAATCAGCTCCCCAAAGCGTCGCTGCG

TGGCCTGCTGATTAAATGCGGCGAGAAACACCGGAAAGTCATGACGGAATAACTCTACCGGAGAGATTTGCGCGGTGGAA

ACGTTCTCTCCGGCTCGCAGCAGGTAAAAGTTGTCCCATGCAGTGTCAGGAGTAGGGTTTAGCATTGTTAACAGCGTGTC

AGTGGAGAGCATCATCGCCGTGTCTGAATCTTGGGAGGAAAGCAGCAGGAAATTATCTGCAGCGCGGGTTGTCCAGTCCG

GGCTGCCGTCATAATTGCCGAAGAGCCCATTATGGATGTGGGGAGACACCGCCGGGTGAGCAAGATACTGTTCATAAAGC

GCTGCAGCTTTCTGGCGGGTGCCATCAGTGCAGTCCACGCCTGTCATACAGTGTAAAATCACCTGATTAAAGAGTCCGCT

GTGAGTGGTCATGGCGGCGCTGTTCCTGTCAAACGAATCCAGCAACACAGGCAGTATGCGCTGCTGATAATATGTGTCAG

GAGTCGGCAGGGGAACGTCATTATAGGTCTGGCACTGGTGGGCAAAAAAACGTTCCTGAACAGGCCCGAGCCACTCGCGG

ATAAGCGCACTTTGCGTATAGGGAGGGTGGGCCAGCACGCTTTACCCAGAAAAAACCTATATTGACGTTGCCCCTTGCCT

CGACTTCTGACCCAAATAATCAGCAGAGCAAAACGGTCGGGTTGTTGTGTATTATCTTTCATAGAGAAACACCTCTCTTT

CTTACTTCAAGCTTTTCTATGTATTGTATTCTCTTCATCGAGGAGGGCTGAG

>24-sbon-3

CTAAGTCTGGGTCAAATGGTGGCAGACGCGACACTGAGGCATCATCAATCAACTTCACGGTGGAATGTTTCTGGTTCGTG

GCGGCAATAAACTGCTGGTTCAGCTCCCCGTGCTCTTCAGTGCTGAGGATGATATCAATCAGCTCCCCAAAGCGTCGCTG

CGTGGCCTGCTGATTAAATGCGGCGAGAAACACCGGAAAGTCATGACGGAATAACTCTACCGGAGAGATTTGCGCGGTGG

AAACGTTCTCTCCGGCTCGCAGCAGGTAAAAGTTGTCCCATGCAGTGTCAGGAGTAGGGTTTAGCATTGTTAACAGCGTG

TCAGTGGAGAGCATCATCGCCGTGTCTGAATCCTGGGAGGAAAGCAGCAGGAAATTATCTGCAGCGCGGGTTGTCCAGTC

CGGGCTGCCGTCATAATTGCCGAAGAGCCCATTATGGATGTGGGGAGACACCGCCGGGTGAGCAAGATACTGTTCATAAA

GCGCTGCAGCTTTCTGGCGGGTGCCATCAGTGCAGTCCACGCCTGTCATACAGTGTAAAATCACCTGATTAAAGAGTCCG

CTGTGATTAGTCATGGCGGCGCTGTTCCTGTCAAACGAATCCAGCAACACAGGCAGTATGCGCTGCTGATAATATGTGTC

AGGAGCCGGCAGGGGAACGTCATTATAGTTTTTGGCACTGGTGGGCAATAAAACGTTTCCTGATCAGGTCCGTTCCTCTT

CGCTGATAAGTGTACTTTGTTGTATAC

>24-sbon-4

CCCAAATGGGTCAAAATGGTGGCAGACGCGACACTGAGGCATCATCAATCAACTTCACGGTGGAATGTTTCTGGTTCGTG

GCGGCAAGAAACTGCTGGTTCAGCTCCCCGTGCTCTTCAGTGCTGAGGATGATATCAATCAGCTCCCCAAAGCGTCGCTG

CGTGGCCTGCTGATTAAATGCGGCGAGAAACACCGGAAAGTCATGACGGAATAACTCTACCGGAGAGATTTGCGCGGTGG

AAACGTTCTCTCCGGCTCGCAGCAGGTAAAAGTTGTCCCATGCAGTGTCAGGAGTAGGGTTTAGCATTGTTAACAGCGTG

TCAGTGGAGAGCATCATCGCCGTGTCTGAATCTTGGGAGGAAAGCAGCAGGAAATTATCTGCAGCGCGGGTTGTCCAGTC

CGGGCTGCCATCATAATTGCCGAAGAGCCCATTATGGATGTGGGGAGACACCGCCGGGTGAGCAAGATACTGTTCATAAA

GCGCTGCAGCTTTCTGGCGGGTGCCATCAGTGCAGTCCACGCCTGTCATACAGTGTAAAATCACCTGATTAAAGAGTCCG

CTGTGAGTGGTCATGGCGGCGCTGTTCCTGTCAAACGAATCCAGCAACACAGGCAGTATGCGCTGCTGATAATATGTGTC

AGGAGCCGGCAGGGGAACGTCATTATAGGTCTGGCACTGGTGGGCAAAAAAAACGTTCCCTGAACAGGCCCGAGCCACTC

GCTGATTAAGCGCACTTTGCGTATAGGGGGGGGTGTGCCAGCACGCTTACCAGATTACAACCATTCTGCCGTGTACCTTG

CTGGTTCCTTGTCCCAATTGTAACGCCTGGACACCGTTTGTTTACTCCTTTGCTCCCTGAAGTACAACCCCCCCCGCCCT

CTCCTTTCTAATTCTTTTTCTCT

>24-sbon-5

AATGTCTTTAGTCAAATGGTGTTCAGACGCGACACTGAGGCATCATCAATCAACTTCACGGTGGAATGTTTCTGGGTCGT

GGCGGCAATAAACTGCTGGTTCAGCTCCCCGTGCTCTTCAGCGCTGAGGATGATATCAATCAGTTCCCCAAAGCGTCGCT

GCACGGCCTGCTGATTAAATGCGGCGAGAAACACCGGAAAGTCATGACGGAATAACTCTACCGGAGAGATTTGCGCGGTG

GAAACGTTCTCTCCGGCTCGCAGCAGGTAAAAGTTGTCCCATGCAGTGTCAGGAGTAGGGTTTAGCATTGTTAACAGCGT

GTCAGTGGAGAGCATCATCGCCGTGTCTGAATCCTGGGAGGAAAGCAGCAGGAAATTATCTGCAGCGCGGGTTGTCCAGT

CCGGGCTGCCGTCATAATTGCCGAAGAGCCCATTATGGATGTGGGGAGACACCGTTGGGTGAGCAAGATATCGTTCATAA

AGCGCTGCAGCTTTCTGGCGGGTGCCATCAGTGCAGCCCACGCCTGTCATACAGTGTAAAATCACCTGATTAAAGAGTCC

GTTGTGAGTGGTCATGGCGGCGTTGTTCCTGTCAAACGAATCCAGCAACACAGGCCGTATGCGCTGCTGATAATATGTGT

CAGGAGCCGGCAGGGGAACGTCATTATAGGTCTGGCACTGGTGGGCAAAAAAAAACGTTCCCTGAACAGGCCCGAACCAC

TCGCTGATAAAGCGTACTTCTGCGGTATAGGGGGGGGTTCAGCACCGCTTACCCAGAAAAAAACTATTAATGGAGGGTTC

CCCCTTGCCGGTTTTCTGACCTATTTTAACCACATTCAATTACCGTTCTCTTTCTGTGTGATTCTTTATAGAACACAC

>24-sbon-6

GAACTGGTATTCGTTCAAATGGTGGCAGACGCGACACTGAGGCATCATCAATCAACTTCACGGTGGAATGTTTCTGGTTC

GTGGCGGCAATAAACTGCTGGTTCAGCTCCCCGTTATCTTCAGTGCTGAGGATGATATCAATCAGCTCCCCAAAGCGTCG

CTGCGTGGCCTGCTGATTAAATGCGGCGAGAAACACCGGAAAGTCATGACGGAATAACTCTACTGGAGAGATTTGCGCGG

TAGAAACGTTCTCTCCGGCTCGCAGCAGGTAAAAGTTGTCCCATGCAGTGTCAGGAGTAGGGTTTAGCATTGTTAACAGC

GTGTCAGTGGAGAGCATCATCGCCGTGTCTGAATCCTGGGAGGAAAGCAGCAGGAAATTATCTGCGGCGCGGGTTGTCCA

GTCCGGGCTGCCGTCATAATTGCCGAAGAGCCCATTATGGATGTGGGGAGACACCGCCGGGTGAGCAATATACTGTTTAT

AATGCGCTGCAGCTTTCTGGGGGGTGCCATCAATGCAATACCACACCTGTTATTTTTTTTTTTTTTTTTTTTTTTATTA

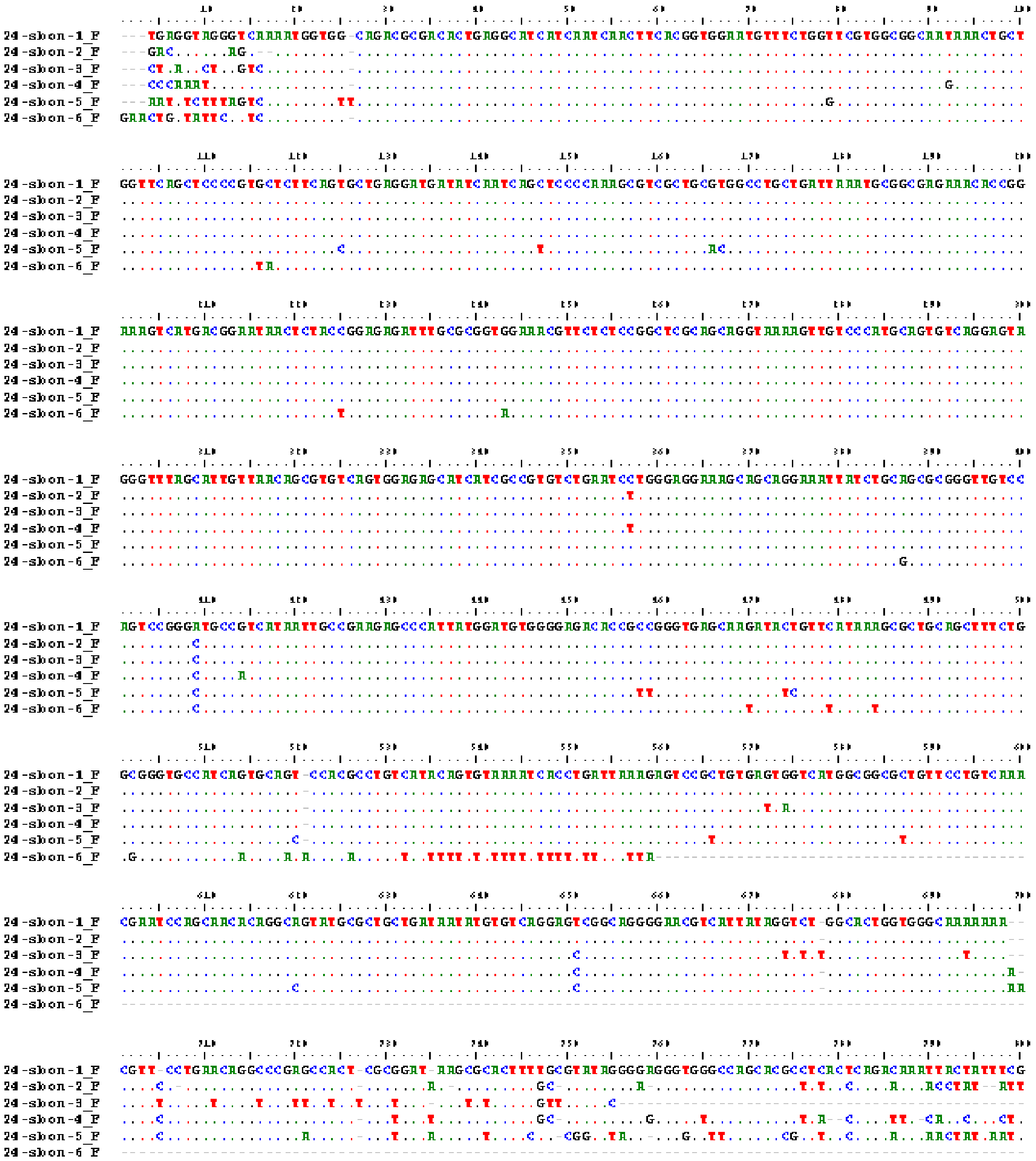

SBG(2)_1F : TTT CTG GTT CGT GGC GGC AAT

SBG(2)_1R : TCT TCG GCA ATT ATG ACG GTA T

SBG(2)_2F : TTT CTG GTT CGT GGC GGC GAT

SBG(2)_2R : TTC CTG CTG CTT TCC TCC TAA

SBG(2)_4F : TTC TGG TTC GTG GCG GCG AG

SBG(2)_4R : ATG GGC TCT TCG GCA ATT ATA AT

SBG(2)_5F : TTC CCC AAA GCG TCG CTG TAC

SBG(2)_5R : GCT GCA GCG CTT TAT GAG CGA

SBG(2)_6F : GCT GGT TCA GCT CCC CAT TA

SBG(2)_6R : CGG ACT GGA CAA CCC GCT CC

The PCR amplification of 2nd time with the primer sets (11-, 12-, 13-, 14-, 15-, 16-, 18-, 19-, 21-Sbon) are as following:

11-Sbon, Gene Name: tRNA (Ile)-lysidine synthase (*mesJ*

**11-Sbon-F**	**TTCTGGCCAGCGACCTTG**
**11-Sbon-R**	**TGCCAGTTTCAGCCACCC**

>11-Sbon-3

GGTGACGAGGATTGCGGCTTTTTCCTTTATTGTCCGATGAAGCGATGTACGGCGGGCGGCGATTTGCGTCGCTGGCTGGC

GATGCGTAATGCGCCGATGCTCTCTCGCGACGCGCTGGAACGTATCTGGCAGGAGGTGGCGCTGGCGCGCGACGATGCCT

CTCCCTGTTTACGTTTTGGCGATCGCGAGATTCGCCGTTATCAGTCGCAACTGTGGTGGATTAAATCCGTGGCCGGACAA

CACGAAACGACGGTCGCCTGGCCCGTCTGGCAAACACCGCTGGCGCTGCCTGCCGGCTTAGGGACGGTACAGCTCGTTCC

TGGCGGCGAACTGCGCCGACCGCGAGAGGAAGAATCCGTCAGCATTCGGTTTAAGGCGCCAGGGTTGCTACACATTGTGG

GACGTCACGGCGGACGTAAATTAAAGAAAATCTGGCAGGAGCAGGGGATCCCGCCCCTGGCGACGAGACACCACGCCGCTG

TTGTTTTACGGCGAAACGCTGATTGCGGCTGCGGGTGTTTTTGTGACGCGTGAGGGAGCAGCAGAAGATAAAGAAGGGGT

GAGTCTGGTTTGGCATGCCTGATGGCGCTGCGCTTATCAGGCCTACGTATCGCATGGAACGTAGGCCGGATAAGGTGCTT

GCACCACCATCCGGCAACAAAATACTCAGGATTCGCTAACTACCACGGTACCAATTTCCGGGTGGTGAAAAACTGGGCAA

>11-Sbon-4

GGCTTTCGGAAGGATTGCGGCTCTCTCCTTTATGTCGATGAAGCGATGTACGGCGGGCGGCGATTTTGCGTCGCTTGGCT

TGGCGATGCGTAATGCGCCGATGCCCTCTCGCGACGCGCTGGAACGTATCTGGCAGGAGGTGGCGCTGGCGCGCGACGAT

GCCTCTCCCTGTTTACGCTTTGGCGATCACGAGATTCGCCGTTACCAGTCGCAACTGTGGTGGATTAAATCCGTGGCCGG

ACAACACGAAACGACGGTCGCCTGGCCCGTCTGGCAAACACCGCTGGCGCTGCCTGCCGGCTTAGGGACGGTACAGCTCG

TTCCTGGTGGCGAACTGCGCCGACCGCGAGAGGAAGAATCCGTCAGCATTCGTTTTAAAGCGCCAGGCGTGTTACATATT

GTCGGGCGTAACGGTGGACGTAAATTAAAGAAAATCTGGCAGGAGCAGGGGATCCCGCCCCTGGCGACGAGATACCACGCC

GCTGTTGTTTTACGGCGAAACGCTGATTGCGGCTGCGGGTGTTTTTGTGACGCGTGAGGGAGCAGCAGAAGATAAAGAAG

GGGTGAGTCTGGTTTGGCATGCCTGATGGCGCTGCGCTTATCAGGCCTACGTATCGCATGGAACGTAGGCCGGATAAGGT

GCTTGCACCACCATCCGGCAACAAAATACTCAGGATTCGCTAACTACCACGGTACCAATTTCCGGGTGCGAAAAACCTGG

CCACGGCCGGATGTCCGGCATCCGATCCCATGCCCACTTCGCCGCATCACCGAACAGCATTCCATGCTCTTCCATCCATG

CCTCATATTGTCGTGTACGTCCCCCATCCGCTTATTGCATTTTCTTACGATTACGTGGTACATGGCTATTTCTCTGCTGA

CGATCAGTCTA

>11-Sbon-5

CTTTACGAGTAATTGCGGCATTACTTATGTCGACAGCGAGTGCGGCGGCGGCGACTTGCGCGCTGGTTGGCGAGCGAAAG

CGTCATCTTATCGCGAGCGCTGGAACGTACTGGCAGGAGGTGGCGCTGGCGCGCGAGATCCCTCTTGTTTACGTTTGGCG

AGCACGAGAGTCGCGTTATCAGTCGCAATGTGGTGGATAAAACCGTACCGGACAACACGAAACGACGGTCGCCTGGCCCG

TCTGGCAAACACCGCTGGCGCTGCTGCCGGTTAGGGACGGTACAGCTCGTTCCTGGCGGCGAATGCGCCGACCGCGAGAG

GAAGATCCGTCAGCATTCGGTTTAAAGCGCCGGGTTGCTACACATTGTGGGACGTCACGGCGGACGTAAATTAAAGAAAA

TCTGGCAGGAGCAGGGGATCCCGCCCTGGCGACGAGACACCACGCCGCTGTTGTTTTACGGCGAACGCTGATTGCGGCTG

CGGGTGTTTTTGTGACGCGTGAGGGAGCAGCAGAAGATAAAGAAGGGTGAGTCTGGTTTGGCATGCCTGATGGCGCTGCG

CTTATCAGGCCTACGTATCGCATGGAACGTAGCCGGATAAGGTGCTTGCACCACCATCCCGGCAACAAAATACTCAGGAT

TCGCTAACTACCACGGTACCATTTCCGGTGGAAAAATGGGAAA

>11-Sbon-6

CCGGATACGAGTAAGTGCGGCATTACTCAATGTCGATGAAGCGATGACGGCGGGCGCGTTTTGCGTCGTTGGTTGGCGAT

GCGTAATGCGTCGACCTTACTCGCGACGCGCTGGAACGTATCTGGCAGGAGGTGGCGCTGGCGCGCGACGATCTTCTCTT

GTTTACGTTTTGGCGATCGCGAGAGTCGCCGTATCAGTCGAACTGTGGTGGATTAAATCCGTGGCCGGACAACACGAAAC

GACGGTCGCCTGGCCCGTCTGGCAAACACCGCTGGCGCTGCTGCCGGCTTAGGGACGGTACAGCTCGTCCTGGCGGCGAA

CTGCGCCGACCGCGAGAGGAAAATCCTCAGCATTCGGTTAAAGCGCCGGCGTATTACATATTGTCGGGCGTAACGGGGAC

GTAATTAAGAAATCTGGCAGGACAGGGGATCCCGCCCTGGCACAGTACCACGCCCTTTGTTTACGGCAACGCTATTGCGG

CTGCGGTGTTTTGTGACGCGTGAGGGACACAAAATAGAAGGGTGATCTGGTTGCATGCCTGATGGCGCTGCGCTTATCAG

CCTACTATCGCTGGACGAGCGGAAAGGTTGCCCCCTCCGGCACAATACTCAGATCCTACTACCACGTCCTTTCCGTGAA

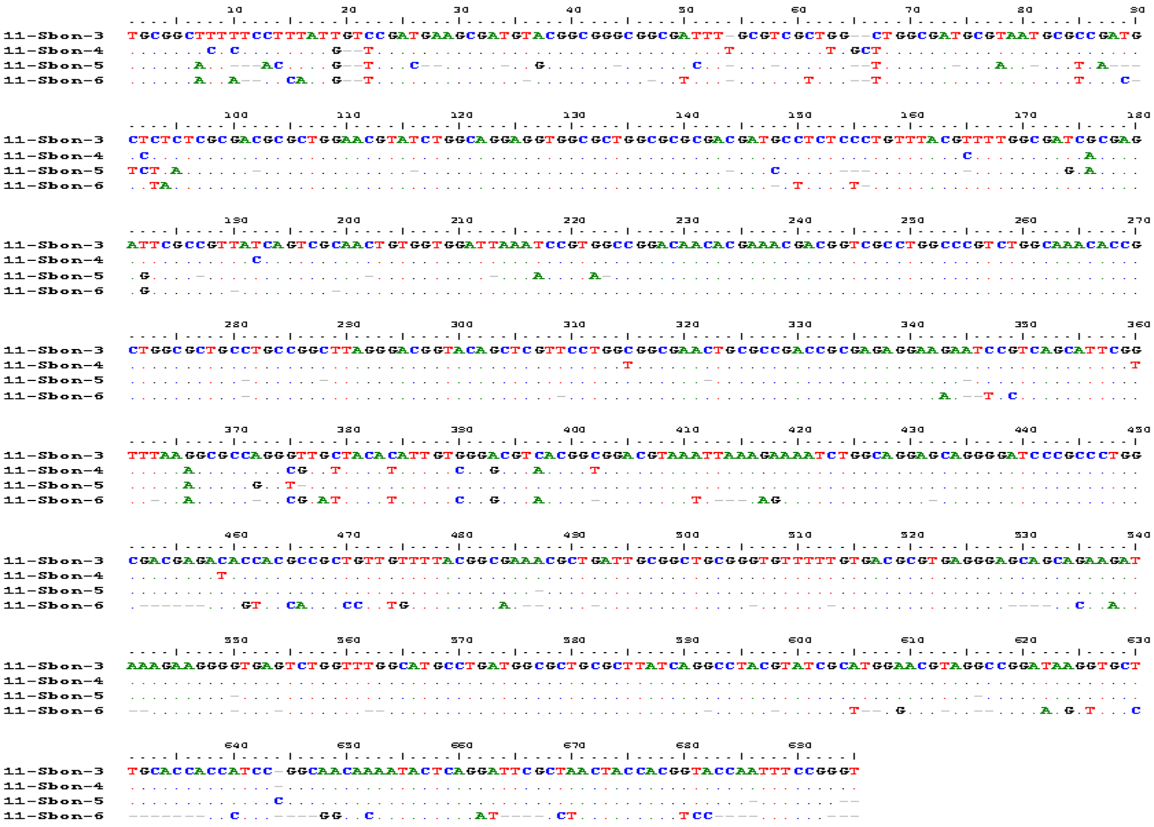

The above alignment of gene, tRNA(Ile)-lysidine synthase (mesJ) did not produce all target sequences of Salmonella, where we did not find any suitable SNPs.

12-Sbon, Gene Name: methionine import ATP-binding protein (*metN*)

**12-Sbon-F**	**ATTGGCACGCTGTCAGCT**
**12-Sbon-R**	**TGCCGGTAAAAGCACGCT**

>12-Sbon-1

GATGCCTATAGCGCCTGGTATCTTCCGGATGTCCAGGTGCAGAGTGGACTGAATAAACTTCTGCGCCAGCGGCGTTTTAG

GGTGCGAGAAGACTTCGCTGACCGTATCCTGCTCAATAAGCTCGCCATTACTGATGACGGCAACGCAGTCGCAGATACGT

TTTACGACATCCATTTCATGAGTGATAAGCAGAATCGTCAACCCCAGACGACGGTTGATATCTTTCAGCAATTCCAGAAT

CGAACGGGTCGTTGCGGGATCAAGCGCGCTGGTGGCTTCATCGCACAGCAACACTTTTGGATTACTGGCTAACGCGCGGG

CAATCGCGACACGCTGTTTCTGACCGCCGGAAAGATTTGCCGGATAGCTGTCGTGCTTGTCGCCAAGACCGACTAAATCC

AGCAGCTCTGTGACACGGCGTTTGATCTCTTCCTTTGGTGTGTTATCCAGTTCCAGCGGCAACGCGACGTTGCCAAACAC

GGTACGGGAAGAGAGCAGGTTAAAATGTTGGAAAATCATGCCGATTTGACGGCGAGCTTTGGTCAACTCAGACTCTGAAA

GCGTTGTCAGCTCCTGACCGCCGACCTGAACGCTACCTTCGGTTGGGCGTTCAAGTAAGTTAACGCAGCGGATAAGCGTG

TTTTTACCCGCAAAAAATTTCCGGGGGTCAAGAACTCGCTCTGCTCCCCGCTTGGCCCGTTTTCGTGGGATATAGTACC

CAAAATGTCTTTGAGCAATGAAACACCTAACGCTAGATCCCGATTCGACGTGTTGCATACTTGTACTTGCAGAGGTGGTC

AACCGTCTCCTGGGGTGACTATCCTACTATTATTCATGCAGTGGAATGCCGTAGTTACTTACCTG

>12-Sbon-2

GAGGCTTACCGCGCTGGTATCTTCCGGATGTCCAGGTGCAGAGTGGACTGAATAAACTTCTGCGCCAGCGGCGTTTTAGG

GTGCGAGAAGACTTCGCTGACCGTATCTTGCTCAATAAGCTCGCCATTACTGATAACGGCAACGCAGTCGCAGATACGTT

TTACGACATCCATTTCATGAGTGATAAGCAGAATCGTCAACCCCAGACGACGGTTGATATCTTTCAGCAATTCCAGAATC

GAACGGGTCGTCGCGGGATCAAGCGCGCTGGTGGCTTCATCGCACAGCAACACTTTTGGATTACTGGCTAACGCGCGGGC

AATCGCGACACGCTGTTTCTGACCGCCGGAAAGATTTGCCGGATAGCTGTCGTGCTTGTCGCCAAGACCGACTAAATCCA

GCAGCTCTGTGACACGGCGTTTGATCTCTTCCTTTGGTGTGTTATCCAGTTCCAGCGGCAACGCGACGTTGCCAAACACG

GTACGGGAAGAGAGCAGGTTAAAATGTTGGAAAATCATGCCGATTTGACGGCGAGCTTTGGTCAACTCAGACTCTGAAAG

CGTTGTCAGCTCCTGACCGCCGACCTGAACGCTACCTTCGGTTGGACGTTCAAGTAAGTTAACGCAGCGGATAAGCGTGT

TTTTACCCGGCA

>12-Sbon-3

GTGGCTTACGGCGCTGGTATCTTCCGGATGTCCAGATGCAGAGTGGACTGAATAAACTTCTGCGCCAGCGGCGTTTTAGG

GTGCGAGAAGACTTCGCTGACCGTATCTTGCTCAATAAGCTCACCATTACTGATGACGGCAACGCAGTCGCAGATACGTT

TTACGACATCCATTTCATGAGTGATAAGCAGAATCGTCAACCCCAGACGACGGTTGATATCTTTCAGCAATTCCAGAATC

GAACGGGTCGTCGCGGGATCAAGCGCGCTGGTGGCTTCATCGCACAGCAACACTTTTGGATTACTGGCTAACGCGCGGGC

AATCGCGACACGCTGTTTCTGACCGCCGGAAAGATTTGCCGGATAGCTGTCGTGCTTGTCGCCAAGACCGACTAAATCCA

GCAGCTCTGTGACACGGCGTTTGATCTCTTCCTTTGGTGTGTTATCCAGTTCCAGCGGCAACGCGACGTTGCCAAACACG

GTACGGGAAGAGAGCAGGTTAAAATGTTGGAAAATCATGCCGATTTGACGGCGAGCTTTGGTCAACTCAGACTCTGAAAG

CGTTGTCAGCTCCTGACCGCCGACCTGAACGCTACCTTCGGTTGGGCGTTCAAGTAAGTTAACGCAGCGGATAAGCGTGC

TTTTACCGGCAA

>12-Sbon-4

TATGCGCTACCGCGCTGGTATCTTCCGGATGTCCAGATGCAGAGTGGACTGAATAAACTTCTGCGCCAGCGGCGTTTTAG

GATGCGAGAAGACTTCGCTGACCGTATCCTGCTCAATAAGCTCGCCATTACTGATAACGGCAACGCAGTCGCAGATACGT

TTCACGACATCCATTTCATGAGTGATAAGCAGAATCGTCAACCCCAGACGACGGTTGATATCTTTCAGCAATTCCAAAAT

CGAACGGGTCGTTGCGGGATCAAGCGCGCTGGTGGCTTCATCGCACAGCAACACTTTTGGATTACTGGCTAACGCGCGGG

CAATCGCGACACGCTGTTTCTGACCGCCGGAAAGATTTGCCGGATAGCTGTCGTGCTTGTCGCCAAGACCGACTAAATCC

AGCAGCTCTGTGACACGGCGTTTGATCTCTTCCTTTGGTGTGTTATCCAGTTCCAGCGGCAACGCGACGTTGCCAAACAC

GGTACGGGAAGAGAGCAGGTTAAAATGTTGGAAAATCATGCCGATTTGACGGCGAGCTTTGGTCAACTCAGACTCTGAAA

GCGTTGTTAGCTCCTGACCGCCGACCTGAACGCTACCTTCGGTTGGGCGTTCAAGTAAGTTAACGCAGCGGATAAGCGTG

TTTTTTACCGGCAAATATTTCCGGGCGGTTATAATCTGCCGGTTCCCGTTGCCCGGTTTGGTGGCCGTTTTTTCTTCTAG

TTTTTCTGTTGCGATGAAAACCCCCTTGC

>12-Sbon-5

GAAGCATTCCGCGCTGGTATCTTCCGGATGTCCAGATGCAGAGTGGACTGAATAAACTTCTGCGCCAGCGGCGTTTTAGG

ATGCGAGAAGACTTCGCTGACCGTATCCTGCTCAATAAGCTCGCCATTACTGATGACGGCAACGCAGTCGCAGATACGTT

TTACGACATCCATTTCATGAGTGATAAGCAGAATCGTCAACCCCAGACGACGGTTGATATCTTTCAGCAATTCCAGAATC

GAACGGGTCGTCGCGGGATCAAGCGCGCTGGTGGCTTCATCGCACAGCAACACTTTTGGATTACTGGCTAACGCGCGGGC

AATCGCGACACGCTGTTTCTGACCGCCGGAAAGATTTGCCGGATAGCTGTCGTGCTTGTCGCCAAGACCGACTAAATCCA

GCAGCTCTGTGACACGGCGTTTGATCTCTTCCTTTGGTGTGTTATCCAGTTCCAGCGGCAACGCGACGTTGCCAAACACG

GTACGGGAAGAGAGCAGGTTAAAATGTTGGAAAATCATGCCGATTTGACGGCGAGCTTTGGTCAACCCGGACTCTGAAAG

CGTTGTCAGCTCCTGACCGCCTACCATTACGCTTCCTTCAGTAGGACGTTCAAGTAAGTTTACGCAGCGAATGAGCGTGC

TTTTTACCGGCAA

>12-Sbon-6

GAGGCTTTCACGCGCCTGGTATCTTCGGATGTCCAGGTGCAGAGTGGACTGAATAAACTTCTGCGCCAGCGGCGTTTTAG

GATGCGAGAAGACTTCGCTGACCGTATCCTGCTCAATAAGCTCGCCATTACTGATAACGGCAACGCAGTCGCAGATACGT

TTCACGACATCCATTTCATGAGTGATAAGCAGAATCGTCAACCCCAGACGACGGTTGATATCTTTCAGCAATTCCAGAAT

CGAACGGGTCGTCGCGGGATCAAGCGCGCTGGTGGCTTCATCGCACAGCAATACTTTTGGATTGCTGGCTAACGCGCGGG

CAATCGCGACACGCTGTTTCTGACCGCCGGAAAGATTTGCCGGATAGCTGTCGTGCTTGTCGCCAAGACCGACTAAATCC

AGCAGTTCTGTGACACGGCGTTTGATCTCTTCCTTTGGTGTGTTATCCAGTTCCAGCGGCAACGCGACGTTGCCAAACAC

GGTACGGGAAGAGAGCAGGTTAAAATGTTGGAAAATCATGCCGATTTGACGGCGAGCTTTGGTCAACCCGGACTCTGAAA

GCGTTGTCAGCTCCTGACCGCCTACCATTACGCTTCCTTCAGTAGGACGTTCAAGTAAGTTTACGCAGCGAATGAGCGTG

TTTTTACCGGCAAA

```
                   10        20        30        40        50        60        70        80        90
12-Sbon-1  CGCCTGGTATCTTCCGGATGTCCAGGTGCAGAGTGGACTGAATAAACTTCTGCGCCAGCGGCGTTTTAGGGTGCGAGAAGACTTCGCTGA
12-Sbon-2  ...-......................................................................................
12-Sbon-3  ...-.....................A................................................................
12-Sbon-4  ...-.....................A............................................A...................
12-Sbon-5  ...-.....................A............................................A...................
12-Sbon-6  ..............-.......................................................A...................

                  100       110       120       130       140       150       160       170       180
12-Sbon-1  CCGTATCCTGCTCAATAAGCTCGCCATTACTGATGACGGCAACGCAGTCGCAGATACGTTTTACGACATCCATTTCATGAGTGATAAGCA
12-Sbon-2  .......T..........................A.......................................................
12-Sbon-3  .......T..............A...................................................................
12-Sbon-4  ..................................A..........................C............................
12-Sbon-5  ..........................................................................................
12-Sbon-6  ..................................A..........................C............................

                  190       200       210       220       230       240       250       260       270
12-Sbon-1  GAATCGTCAACCCCAGACGACGGTTGATATCTTTCAGCAATTCCAGAATCGAACGGGTCGTTGCGGGATCAAGCGCGCTGGTGGCTTCAT
12-Sbon-2  .............................................................C............................
12-Sbon-3  .............................................................C............................
12-Sbon-4  .............................................A............................................
12-Sbon-5  .............................................................C............................
12-Sbon-6  .............................................................C............................

                  280       290       300       310       320       330       340       350       360
12-Sbon-1  CGCACAGCAACACTTTTGGATTACTGGCTAACGCGCGGGCAATCGCGACACGCTGTTTCTGACCGCCGGAAAGATTTGCCGGATAGCTGT
12-Sbon-2  ..........................................................................................
12-Sbon-3  ..........................................................................................
12-Sbon-4  ..........................................................................................
12-Sbon-5  ..........................................................................................
12-Sbon-6  ..........T...........G...................................................................

                  370       380       390       400       410       420       430       440       450
12-Sbon-1  CGTGCTTGTCGCCAAGACCGACTAAATCCAGCAGCTCTGTGACACGGCGTTTGATCTCTTCCTTTGGTGTGTTATCCAGTTCCAGCGGCA
12-Sbon-2  ..........................................................................................
12-Sbon-3  ..........................................................................................
12-Sbon-4  ..........................................................................................
12-Sbon-5  ..........................................................................................
12-Sbon-6  ..................................T.......................................................

                  460       470       480       490       500       510       520       530       540
12-Sbon-1  ACGCGACGTTGCCAAACACGGTACGGGAAGAGAGCAGGTTAAAATGTTGGAAAATCATGCCGATTTGACGGCGAGCTTTGGTCAACTCAG
12-Sbon-2  ..........................................................................................
12-Sbon-3  ..........................................................................................
12-Sbon-4  ..........................................................................................
12-Sbon-5  ......................................................................................C.G.
12-Sbon-6  ......................................................................................C.G.

                  550       560       570       580       590       600       610       620       630
12-Sbon-1  ACTCTGAAAGCGTTGTCAGCTCCTGACCGCCGACCTGAACGCTACCTTCGGTTGGGCGTTCAAGTAAGTTAACGCAGCGGATAAGCGTGT
12-Sbon-2  .......................................................A..................................
12-Sbon-3  .........................................................................................C
12-Sbon-4  ................T.........................................................................
12-Sbon-5  ...............................T...ATT.....T.....A..A..A..............T........A..G......C
12-Sbon-6  ...............................T...ATT.....T.....A..A..A..............T........A..G......-

                  640
12-Sbon-1  TTTTTACCCGCA
12-Sbon-2  ....AC..G...
12-Sbon-3  ....-...G...
12-Sbon-4  ........G...
12-Sbon-5  ........G...
12-Sbon-6  ........G...
```

The designed SNP-based primers are based on the above align gene (*metN*) sequences (natural SNP is marked by red color and artificial mutated bases are marked by yellow shaded)

metN_F1: GACTTCGCTGACCGTACCC

metN_R1: GCT GCG TTA ACT TAC TTG AAT GC

metN_F3: CCG TAT CTT GCT CAA TAA GCC CA

metN_R3: GCC CAA CCG AAG GTA GCG T

metN_F4: GGT TGA TAT CTT TCA GCA ATT CTA A

metN_R4: GGT CGG CGG TCA GGA GTT A

metN_F5: CGC ACA GCA ACA CTT TTG GAT TA

metN_R5: CCT ACT GAA GGA AGC GTA ATG ATA

metN_F6: CGC ACA GCA ATA CTT TTG GAC TG

metN_R6: CCT ACT GAA GGA AGC GTA ATG ATA

13-Sbon, Gene Name: Pyrimidine-specific ribonucleoside hydrolase (*rihB*)

**13-Sbon-F**	**GCTGTACCTGCCGACTGG**
**13-Sbon-R**	**GTTCCCCACGGGCTATGG**

>13-Sbon-1

GAGCGATAGTGTCTGGCAAGTAAATGCATTGCAGCATTAACGAAGAAAGGAAGTGATCTGGTTAACACTGTAATGTAAAA

AATGGGTAACGATACGGTTTTATCATGTTGTTGTGTTGTTAATTACGGTAAAGGTGTTATAGAAACAAAATGTAACATCT

CTCTGGAACACCCAAACGGACAACAACTATGAATAAATCAGGGAAATACCTCGTCTGGACAGCGCTCTCAGTATTGGGTG

CGTTTGCCCTGGGCTATATTGCGTTAAATCGTGGGGAACAGATCAACGCGCTATGGATCGTGGTGGCGTCGGTCTGTGTC

TATCTTATTGCGTATCGTTTTTATGGGCTCTATATCGCCAAAAAAGTGCTGGCGGTTGACCCAACGCGTATGACGCCCGC

GGTACGTCATAATGATGGTCTGGATTATGTCCCGACCGATAAGAAAGTGCTTTTTGGTCACCATTTTGCGGCCATTGCTG

GCGCAGGTCCGCTGGTCGGGCCGGTACTGGCGGCGCAGATGGGCTATCTGCCGGGGATGATCTGGCTGCTGGCGGGCGTC

GTGCTGGCGGGAGCGGTGCAGGACTTTATGGTGCTGTTCGTCTCGACCCGGCGCGATGGGCGTTCGCTTGGCGAGCTGGT

TAAAGAGGAGATGGGCGCGACGGCAGGGGTGATCGCGCTGGTGGCCTGCTTTATGATCATGGTGATCATTCTGGCCGTCC

TGGCGATGATCGTGGTGAAAGCGCTGACCCATAGCCGGGGGGGGGAAACAAACAGCACCATAAAGTCCTGCACCGGCTGT

CTTTCCCGCACGAAAAACACCCCGCCCCCGGGCACATATAATAAATTAATGGCGCAAGATGCCACTTTCCTCTCCGGGTC

CGGTCCGGACAGGAGGATATTTCTCCCCCATTGGGCCCGCAAAAATGGTTAACAAAAAGAGCTATTTTTCTTTAGTCGGG

GGAGGTATATTCATCACACTACCATTCTATATTTAACGGTAGCGCGGCGCGTCAATACGGCGGTTTGGGCGTCCACCCCG

TCTTGCTGTTTTTTTGGGCGATTTACAGCTCAATGAATACCGATCGCAATTATAAATGAACACAGCACCGACCTCCGCGA

CGATTCTTAGTCGGCTGTTGGATTTGTTCCTCACGATTTACGGCATATAGTCTATGGGCTGATACGCCATCGAACGATGG

CGCTTTTCCGAACCTAGGCTATTTCGCCTGTGAACTGTATGATCA

>13-Sbon-2

AACCCTTGGGTTATTTCTGGCAGTAAATGCAATTGCAGCATTAACGAAGAAAGGAAGTGATCTGGTTAACACTGTAATGT

AAAAAATGGGTAACAATCCGGTTTTATCATGTTGTTGTGTTGTTAATTACGGTAAAGGTGTTATAGAAACAAAATGTAAC

ATCTCTCTGGAACACCCAAACGGACAACAACTATGAATAAATCAGGGAAATACCTCGTCTGGACAGCGCTCTCAGTATTG

GGTGCGTTTGCCCTGGGCTATATTGCGTTAAATCGTGGGGAACAGATCAACGCGCTATGGATCGTGGTGGCGTCGGTCTG

TGTCTATCTTATTGCGTATCGTTTTTATGGGCTCTATATCGCCAAAAAAGTGCTGGCGGTTGACCCAACGCGTATGACGC

CCGCGGTACGTCATAATGATGGTCTGGATTATGTCCCGACCGATAAAAAAGTGCTGTTCGGTCACCATTTTGCGGCCATT

GCTGGCGCAGGTCCGCTGGTCGGGCCGGTACTGGCGGCGCAGATGGGCTATCTGCCGGGGATGATCTGGCTGCTGGCGGG

CGTCGTGCTGGCGGGAGCGGTGCAGGACTTTATGGTGCTGTTCGTCTCGACCCGGCGCGATGGGCGTTCGCTTGGCGAGC

TGGTTAAAGAGGAGATGGGCGCGACGGCAGGGGTGATCGCGCTGGTGGCCTGCTTTATGATCATGGTGATCATTCTGGCC

GTCCTGGCGATGATCGTGGTGAAAGCGCTGACCCATAGCCGTGGGGGAACAAACAGCACCATAAAGTCCCTGCACCGGCT

TCTCTCTCCACTGTCTT

>13-Sbon-6

GAGCCTTATTATTGTTCTGGCAAGTAAATGCAATTGCAGCATTAACGAAGAAAGGAAGTGATCTGGTTAACACTGTAATG

TAAAAAATGGGTAACAATCCGGTTTTATCATGTTGTTGTGTTGTTAATTACGGTAAAGGTGTTATAGAAACAAAATGTAA

CATCTCTCTGGAACACCCAAACGGACAACAACTATGAATAAATCAGGGAAATACCTCGTCTGGACAGCGCTCTCAGTATT

GGGTGCGTTTGCCCTGGGCTATATTGCGTTAAATCGTGGGGAACAGATCAACGCGCTATGGATCGTGGTGGCGTCGGTCT

GTGTCTATCTTATTGCGTATCGTTTTTATGGGCTCTATATCGCCAAAAAAGTGCTGGCGGTTGACCCAACGCGTATGACG

CCCGCGGTACGTCATAATGATGGTCTGGATTATGTCCCGACCGATAAGAAAGTGCTTTTTGGTCACCATTTTGCGGCCAT

TGCTGGCGCAGGTCCGCTGGTCGGGCCGGTACTGGCGGCGCAGATGGGCTATCTGCCGGGGATGATCTGGCTGCTGGCGG

GCGTTGTGCTGGCGGGAGCGGTGCAGGACTTTATGGTGCTGTTCGTCTCGACCCGACGCGATGGGCGTTCGCTTGGCGAG

CTGGTTAAAGAGGAGATGGGCGCGACGGCAGGGGTGATCGCCCTGGTGGCCTGCTTTATGATCATGGTGATCATTCTGGC

CGTCCTGGCGATGATCGTGGTGAAAGCGCTGACCCATAGCCCGTGGGGGAAACACACAGCACCATAAAAGTCCTGCACCG

CTTCTTCCCTCTAATATTATTCTTGCTTCTGTACCTTAAAATATCTAACACGGTGTATGCTAATTCCCTCTCCCGTACGT

CCAACCATATGAATTTTCCTCCCATTGGCCCCCAAATGGTTAACAAAAGCTCTTTTTTTTTTGTGGGGTGGTAAATCAAC

CCAATCTATTAAATATTTACCGTAGCCGTGGTGACTCATAAGTCGGTGGGGTCCTCCTGCTTTTATTTTTTATGGTCTAA

TTTGCATCCCCTTTTGTACTGAC

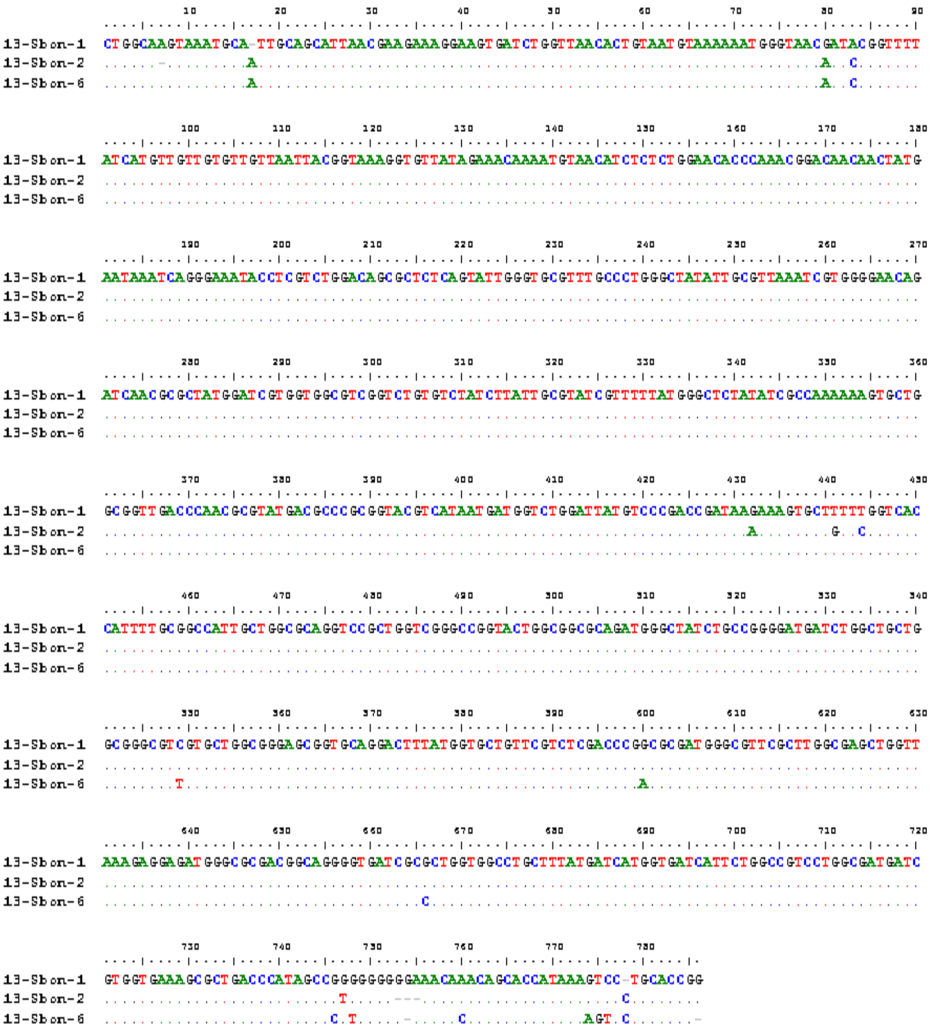

The above alignment of gene(rihB) did not produce the all target sequences of Salmonella, where we did not find any suitable SNPs.

14-Sbon, Pyrimidine-specific ribonucleoside hydrolase (rihA)

**14-Sbon-F**	**TCCCCTGTGTTTCGACGC**
**14-Sbon-R**	**ACGCCGGATAAGACGCTG**

>14-Sbon-1

CGGATGGAAAGGGTGCAATCTCGGGCTGGAGCAGCCAGGCGATGGTGCACGGGTCGTGCAGCGGTGCGCCGACGAATCCC

CATTTTTCGTCTTTGTGGTACTCCATAAAGAAGTCCAGCAGTTCGGCGACGATGGTGGAAATCGGGTTACCGATGGCGCG

AAAGCGTTCGATATCGGCGGCGTGAATCTGTGCCTTATGAGTGACGTCCAGCCCGGCCATCACCACCGGAATGCCTGACT

GGAAGACAATTTCTGCCGCTTCCGGGTCGACGTAAATATTAAATTCCGCAGCGGGCGTCCAGTTACCCAGCGCCATCGCG

CCGCCCATGATCACAATACGGGCGATTTTCGTATGCAGTTCCGGATGGCTATTCAGCAGCAGCGCCACGTTGGTCTGTGG

GCCAGTGGAGACGATGGTGACCGGCTGTGAGCTCTCGCGCAGTGTTTTTGCCATCAGCTCAACGGCAGTGCCGCTTTGCG

GGGCGAATGACGGCTCGGGCAGCGCCGGGCCGTCGAGGCCGCTTTCGCCGTGAACGTTATCGGCGATAATCAGTTCGCGC

ATGAGAGGTTTCACCGCGCCGCCCGCCACCGGAATATCCGGGCGTTTGAGCAGTGTCAGCATCCGCAGCACGTTGCGCAG

CGTCTTATCCGGCGTATGGGGAATTTTTCTTGATTCGCTTACTTTTCTCTGTTTCTTACTTTTCGTGTAATTTTTTTTTG

TGATTTGATTTTTCGATTTATTATGGAGGATCGCCTG

>14-Sbon-3

TTGGGCTTGCGGGGGTTAATCTCGGGTTTCAGCAGCCAGGCGATGGTGCACGGGTCGTGCAGCGGCGCGCCGACAAATCC

CCATTTTTCGTCTTTGTGATACTCCATAAAGAAGTCCAGCAGTTCGGCGACGATGGTGGAAATCGGGTTACCGATGGCGC

GGAAACGCTCGATATCGGCGGCGTGAATCTGCGCCTTATGGGTGACGTCCAGCCCGGCCATCACCACCGGAATGCCTGAC

TGGAAGACAATTTCTGCCGCTTCCGGGTCGACGTAAATATTAATTGTTAATTCCGCAGCGGGCGTCCAGTTCCCCAATCC

CATTGCGCCGCCCATGATCACAATGCGCGCATTTTCGCTGCAGTCCGGTGGTATCGGTATCACACACGACACGCCACGTG

GGCTGGGGCAGGAGGAGAGACGATGGTGACTTGGCTGTGAGCTCTCACGCAGTGTTTTTGCCATCAGCTCAACGGCAGTG

CCGCTTTGCGGGGCGAATGACGGCTCGGGCAGCGCCGGGCCGTCGAGGCCGCTTTCGCCGTGAACGTTATCGGCGATAAT

CAGTTCGCGCATGAGAGGTTTCACCGCACCGCCCGCCACCGGAATATCCGGGCGTTTGAGCAGCGTCAGCATCCGCAGCA

CGTTGCGCAGCGTCTATCCGGCGT

>14-Sbon-4

GGGGTATTTGAGCTGAAATCTCGGGCTTGAGCAGCCAGGCGATGGTGCACGGGTCGTGCAGCGGTGCGCCGACAAATCCC

CATTTTTCGTCTTTGTGGTACTCCATAAAGAAGTCCAGCAGTTCGGCGACGATGGTGGAAATCGGGTTACCGATGGCGCG

AAAGCGTTCGATATCGGCGGCGTGAATCTGTGCCTTATGAGTGACGTCCAGCCCGGCCATCACCACCGGAATGCCTGACT

GGAAGACAATTTCTGCCGCTTCCGGGTCGACGTAAATATTAAATTCCGCAGCGGGCGTCCAGTTACCCAGCGCCATCGCG

CCGCCCATGATCACAATGCGGGCGATTTTCGTATGCAGTTCCGGATGGCTATTCAGCAGCAGCGCCACGTTGGTCTGTGG

GCCAGTGGAGACGATGGTGACCGGTTGTGCGCTCTCGCGCAGCGTTTTTGCCATCAGCTCAACGGCAGTGCCGCTTTGCG

GGGCGAATGACGGCTCGGGCAGCGCCGGGCCGTCGAGGCCGCTTTCGCCGTGAACGTTATCGGCGATAATCAGTTCGCGC

ATGAGAGGTTTCACCGCGCCGCCCGCCACCGGAATATCCAGGCGTTTGAGCAGTGTCAGCATCCGCAGCACGTTGCGCAG

CGTCTTTAATCACGGCGTA

>14-Sbon-6

GGCTTCGACGTGGTGAATCTCAGGTTTCAGCAGCCAGGCGATAGTGCAAGGGTCGTGCAGCGGCGCGCCGACAATCCCCA

TTTTTCGTCTTTGTGGTACTCCATAAAGAAGTCCAGCAGCTCGGCGACGATGGTGGAAATGGGGTTGCCAATGGCGCGAA

AGCGTTCGATGTCGGCGGCGTGAATCTGCGCCTTATGGGTGACGTCCAGCCCGGCCATCACCACCGGAATGCCTGACTGG

AAGACGATTTCCGCCGCTTCGGGGTCGACGAAATGTTGAACTCGGCCGCGGGCGTCCAGTTGCCGAGCGTCATGGCGCCG

CCCATGATCACGATGCGGGCGATTTTTGTATGCAGTTCCGGATGGCTATTCATCAGCAGCGCCCGTTGGTCTGTGGGCCA

GTGGAGACGATGGTGACCGGCTGTGCGCTCTCGCGCAGGGTTTTTGCCATCAGCTCACGGCGTGCCGCTTTGCGGGGCGA

ATGACGGCTCGGGCAGCGCTGGGCCGTCGAGGCCGCTTTCGCCGTGAACGTTATCAGCGATGATCAGTTCGCGCATGAGA

GGTTTCACCGCGCCGCCCGCCACCGGAATATCCGGGCGTTTGAGCAGCGTCAGCATCCGCAGCACGTTGCGCAGCGTCTT

AAACCAAGCGTA

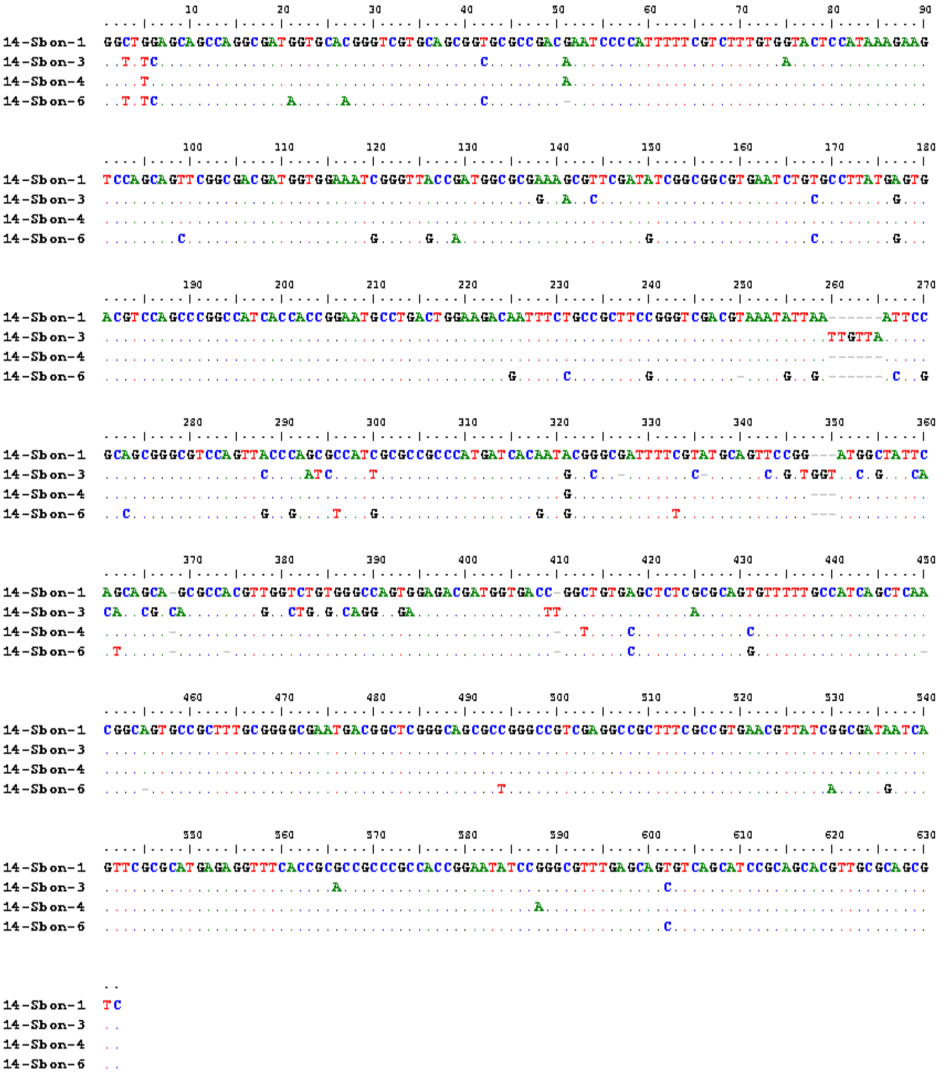

<u>The above alignment of gene (rihA) did not produce the all target sequences of Salmonella, where we did not find any suitable SNPs.</u>

15-Sbon, Gene Name: Molybdate-binding periplasmic protein (*modA*)

**15-Sbon-F**	**GCGGGAAACTCCTGTGCT**
**15-Sbon-R**	**CAACACCCGGCAGCAAAC**

>15-Sbon-1

AAACTGTAAGCGTCGTATCATACCGGTACGCGTTCGCCGCAGTGGCAGCCCCGTCTGGTGTGGAGTTGCCTGGCATTATA

TGTCGTTTTTCTTACCGCGCTGGAGCTCAGGCAAGAGTTATGGGGACTGGTGTTGGTGGCGGCTGGGTTTATTGTGCTGG

CGCGCAGGGTGATAGTCAGCGTGGACTGGACGCTGCTGCTGGTATTTATGGCGATGTTTATTGATGTCCATCTGCTAACG

CAATTGCCGGCGTTGCAGGGCGTTTTCAACCAGGTTGGCGCACTGTCTCACCTGGGGCTTTGGCTAACCGCTATCGGCCT

GTCGCAGGTTATCAGTAATGTGCCCAGTACCATTCTCCTGCTGAACTATGTACCGGCCTCGACGTTGCTGGCGTGGGCGG

TGAATATCGGCGGGTTTGGTTTGCTGCCGGGTGTT

>15-Sbon-2

ATCAAATGTAGGGCGTTCGTATCATACCGGTACGCGTTCGCCGCAGTGGCAGCCCCGTCTGGTGTGGAGTTGCCTGGCAT

TATATGTCGTTTTTCTTACCGCGCTGGAGCTCAGGCAAGAGTTATGGGGACTGGTGTTGGTGGCGGCTGGGTTTATTGTG

CTGGCGCGCAGGGTGATAGTCAGCGTGGACTGGACGCTGCTGCTGGTATTTATGGCGATGTTTATTGATGTCCATCTGCT

AACGCAATTGCCGGCGTTGCAGGGCGTTTTCAACCAGGTTGGCGCACTGTCTCACCTGGGGCTTTGGCTAACCGCTATCG

GCCTGTCGCAGGTTATCAGTAATGTGCCCAGTACCATTCTCCTGCTGAACTATGTACCGGCCTCGACGTTGCTGGCGTGG

GCGGTGAATATCGGCGGGTTTGGTTTGCTGCCGGATGTTGAAGACTACTGAACATCATCGCAATGCTTTTAGCTGCCGGG

TGTTTTGTTTTTTTTTTTTTACTGTTAATACGGTGTGGGGGGGGTGTGTTCAAATTCCCCCCCCCCCCACATATAAAAAC

TTTT

>15-Sbon-3

ATAGCATAGTAGGCGTTAGTATCATACCGGTACGCGTTCGCCGCAGTGGCAGCCCCGTCTGGTGTGGAGTTGCCTGGCAT

TATATGTCGTTTTTCTTACCGCGCTGGAACTCAGGCAAGAGTTATGGGGATTGGTGTTGGTGGCGGCTGGGTTTATTGTG

CTGGCGCGCAGGGTGATAGTCAGCGTGGACTGGACGCTGCTGCTGGTATTTATGGCGATGTTTATTGATGTCCATCTGCT

AACGCAATTGCCGGCGTTGCAGGGCGTTTTCAACCAGGTTGGCGCACTGTCTCACCTGGGACTTTGGCTAACCGCTATCG

GCCTGTCGCAGGTTATCAGTAATGTGCCCAGTACCATTCTCCTGCTGAACTATGTACCGGCCTCGACGCTGCTGGCGTGG

GCGGTGAATATCGGCGGGTTTGGTTTGCTCCCGGTGTTGAAGAAATCGGATGTGGTTGAATTCAGTCAATTTGGTGTATT

TTTTTTTTTTTTTTTTTTTCCCGGTATTAGTTGTGGGGGTGTGTTGTTTATCTCCCCCCCTCCTAGAGTTTTTTTTT

>15-Sbon-4

GGTCTCCCTAGGGCGTTCAGTATCATACCGGTACGCGTTCGCCGCAGTGGCAGCCCCGTCTGGTGTGGAGTTGCCTGGCA

TTATATGTCGTTTTTCTTACCGCGCTGGAGCTCAGGCAAGAGTTATGGGGACTGGTGTTGGTGGCGGCTGGGTTTATTGT

GCTGGCGCGCAGGGTGATAGTCAGCGTGGACTGGACGCTGCTGCTGGTATTTATGGCGATGTTTATTGATGTCCATCTGC

TAACGCAATTGCCGGCGTTGCAGGGCGTTTTCAACCAGGTTGGCGCACTGTCTCACCTGGGGCTTTGGCTAACCGCTATC

GGCCTGTCGCAGATTATCAGTAATGTGCCCAGTACCATTCTCCTGCTGAACTATGTACCGGCCTCGACGCTGCTGGCGTG

GGCGGTGAATATCGGCGGGTTTGGTTTGCTGCCGGGGTGTTGAAGAACTCCCGCACGACGTCTGATTGCCACAATTTGTG

AGTTTTTTTGTTTTTTTTTTTTTTAAATCTTTTGAATGGTTGGGGGGGGGTTGTTTCTCCCCCCCCCCTGTATACTTTTT

TTTT

>15-Sbon-5

GAAGCGGCTAAGGCGTTCGTATCATACCGGTACGCGTTCGCCGCAGTGGCAGCCCCGTCTGGTGTGGAGTTGCCTGGCAT

TATATGTCGTTTTTCTTACCGCGCTGGAGCTCAGGCAAGAGTTATGGGGACTGGTGTTGGTGGCGGCTGGGTTTATTGTG

CTGGCGCGCAGGGTGATAGTCAGCGTGGACTGGACGCTGCTGCTGGTATTTATGGCGATGTTTATTGATGTCCATCTGCT

AACGCAATTGCCGGCGTTGCAGGGCGTTTTCAACCAAGTTGGCGCACTGTCTCACCTGGGACTTTGGCTAACCGCTATCG

GCCTGTCGCAGGTTATCAGTAATGTGCCCAGTACCATTCTCCTGCTGAACTATGTACCGGCCTCGACGTTGCTGGCGTGG

GCGGTGAATATCGGCGGGTTTGGTTTGCTCCCGGGGTGTTGA

>15-Sbon-6

GTGCTGGTTAGGCGTTCGTATCATACCGGTACGCGTTCGCCGCAGTGGCAGCCCCGTCTGGTGTGGAGTTGCCTGGCATT

ATATGTCGTTTTTCTTACCGCGCTGGAGCTCAGGCAAGAGTTATGGGGACTGGTGTTGGTGGCGGCTGGGTTTATTGTGC

TGGCGCGCAGGGTGATAGTCAGCGTGGACTGGACGCTGCTGCTGGTATTTATGGCGATGTTTATTGATGTCCATCTGCTA

ACGCAATTGCCGGCGTTGCAGGGCGTTTTCAACCAGGTTGGCGCACTGTCTCACCTGGGGCTTTGGCTAACCGCTATCGG

CCTGTCGCAGGTTATCAGTAATGTGCCCAGTACCATTCTCCTGCTGAACTATGTACCGGCCTCGACGTTGCTGGCGTGGG

CGGTGAATATCGGCGGGTTTGGTTTGCTGCCGGGTGTTGA

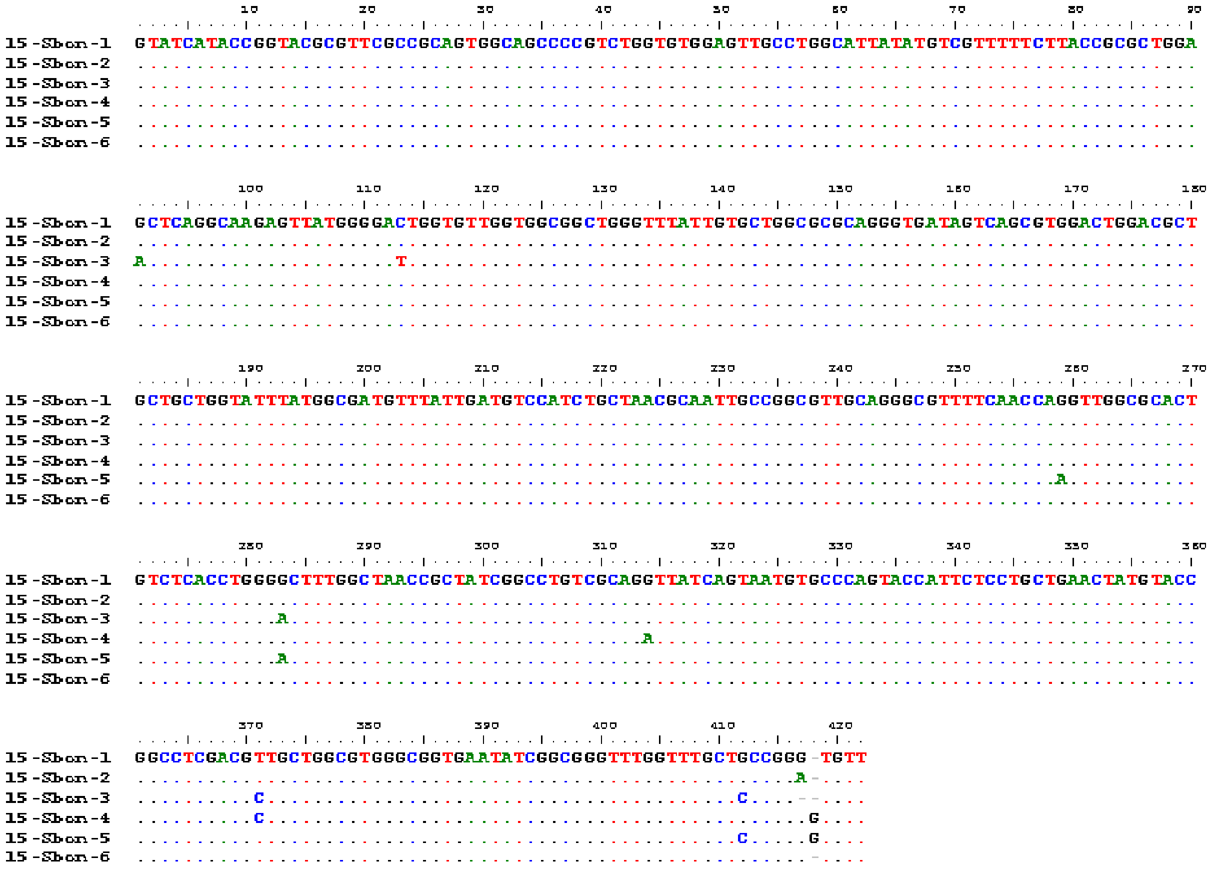

The above alignment of gene(modA) did not produce the all target sequences of Salmonella, where we did not find any suitable SNPs.

16-Sbon, Gene Name: Molybdate-binding periplasmic protein (*modA*)

**16-Sbon-F**	**ACGGTCTGGGTGAGGTGT**
**16-Sbon-R**	**CCACCGCATCAGAACCGT**

>16-Sbon-1

TCAAAACGGTTCGGGTCAGAGACAGTCCAGACAGAACCGCTGATTTCCCCAAACGCCCGCTCAGTCGGGCGTTTTTGTTA

CTCAAGATCCTGCTTTTCCTGCCGATATTTTCTGTGTTTAATCGTTAATGGGTATGAATAACCGCTGGGAAACCCCTGAG

ATTATCGTTATATTGTCGTTTACATAACGAAACACACAAGGAGTTACAGATGGCGCATTCCTGGTTACGCCTGGTCGCAG

GGGCGACATTATCTTTCGTCATCGCTGGTCATGCGCTGGCGGATGAAGGCAAGATCACCGTATTTGCCGCCGCGTCGCTA

ACGAACGCGATGCAAGACATTGCGGCAGAATATAAAAAAGAGAAAAATGTGGATGTGGTCTCCTCATTCGCCTCCTCTTC

AACGCTGGCGCGCCAGATAGAAGCGGGCGCGCCGGCGGATCTGTTCATCTCTGCTGACCAGAAATGGATGGATTACGCAG

CCGATAAAAAAGCGGTGGATACCACGACGCGCGAAACATTGCTTGGCAATCGCCTGGTGGTGGTGGCGCCGAAAGCCAGC

GAGCAGAAACCGTTTACCATCGACAACAAAACGGACTGGATTCGTCTGCTGAACGGCGGACGTCTGGCAGTGGGCGATCC

GCAACACGTACCGGCGGGAATTTATGCCAAAGAAGCGCTGCAAAAGCTGGGCGCATGGCATACGCTTGAACCGAAACTGG

CGCCGGGCGAGGATGTGCGCGGCGCGTTGTGCTGGTCGAACGTAACGAAGCGTCTTTGGGCAT

>16-Sbon-2

CCATACGGGTCGGTCAGAGACAGTCCAGACAGAACCGCTGATTTCCCCAAACGCCCGCTCAGTCGGGCGTTTTTGTTACT

CAAGATCCTGCTTTTCCTGCCGATATTTTCTGTGTTTAATCGTTAATGGGTATGAATAACCGCTGGGAAACCCCTGAGAT

TATCGTTATATTGTCGCTTACATAACGAAACACACAAGGAGTTACAGATGGCGCATTCCTGGTTACGCCTGGTCGCAGGG

GCAACATTATCTTTCGTCATCGCCGGTCATGCGCTGGCGGATGAAGGCAAGATCACCGTATTTGCCGCCGCGTCGCTAAC

GAACGCGATGCAAGACATTGCGGCAGAATATAAAAAAGAGAAAAATGTGGATGTGGTCTCCTCATTCGCCTCCTCTTCAA

CGCTGGCGCGCCAGATAGAAGCGGGCGCGCCGGCGGACCTGTTCATCTCTGCTGATCAGAAATGGATGGATTACGCAGCC

GATAAAAAAGCGGTGGATACCACGACGCGCGAAACATTGCTTGGCAATAGCCTGGTGGTGGTGGCGCCGAAAGCCAGCGA

GCAGAAACCGTTTACCATCGACAACAAAACGGACTGGATTCGTCTGTTGAACGGCGGGCGTCTGGCAGTGGGCGATCCGC

AACACGTACCGGCGGGAATTTATGCCAAAGAAGCGCTGCAAAAGCTGGGCGCATGGCAAACGCTTGAACCGAAACTGGCG

CCGGGCGAGGATGTGCGCGGCGCGTTGGCGCTGGTCGAACGTAACGAAGCGCCTTTGGGCATTGTATACGGTTCGATAGG

CCGGGTGGGAA

>16-Sbon-3

CAAATTGGTACGGTCAGAAGACCAGTCCAGACAGAACCGCTGATTTCCCCAAACGCCCGCTCAGTCGGGCGTTTTTGTTA

CTCAAGATCCTGCTTTTCCTGCCGATATTTTCTGTGTTTAATCGTTAATGGGTATGAATAACCGCTGGGAAACCCCTGAG

ATTATCGTTATATTATCGTTTACATAACGAAACGCACAAGGAGTTACAGATGGCGCATTCCTGGTTACGCCTGGTCGCAG

GGGCGACATTATCTTTCATCATCGCTGGTCATGCGCTGGCGGATGAAGGCAAGATCACCGTATTCGCCGCCGCGTCGCTA

ACGAACGCGATGCAAGACATTGCGGCAGAATATAAAAAAGAGAAAAATGTGGATGTGGTTTCCTCATTCGCCTCCTCTTC

AACGCTGGCGCGCCAGATAGAAGCGGGCGCGCCGGCGGATCTGTTCATCTCTGCTGACCAGAAATGGATGGATTACGCAG

CCGATAAAAAAGCGGTGGATACCACGACGCGCGAAACATTGCTTGGCAATAGCCTGGTGGTGGTGGCGCCGAAAGCCAGC

GAGCAGAAACCGTTTACCATCGATAACAAAACGGACTGGATTCGTCTGCTGAACGGCGGGCGTCTGGCGGTGGGCGATCC

GCAACACGTACCGGCGGGAATTTATGCCAAAGAAGCGCTGCAAAAGTTGGGCGCATGGCAAACGCTTGAACCGAAACTGG

CGCCGGGCGAGGATGTGCGCGGCGCGTTGGCGCTGGTCGAACGTAACGAAGCGCCTTTGGGCATTGTATACGGTTCGAAA

>16-Sbon-4

CAATTATAGTACGGTCAGAAGACAGTCCAGACAGAACCGCTGATTTCCCCAAACGCCCGCTCAGTCGGGCGTTTTTGTTA

CTCAAGATCCTGCTTTTCCTGCCGATATTTTCTGTGTTTAATCGTTAATGGGTATGAATAACCGCTGGGAAACCCCTGAG

ATTATCGTTATATTATCGTTTACATAACGAAACGCACAAGGAGTTACAGATGGCGCATTCCTGGTTACGCCTGGTCGCAG

GGGCAACATTATCTTTCGTCATCGCCGGTCATGCGCTGGCGGATGAAGGCAAGATCACCGTATTTGCCGCCGCGTCGCTA

ACGAACGCGATGCAAGACATTGCGGCAGAATATAAAAAAGAGAAAAATGTGGATGTGGTCTCCTCATTCGCCTCCTCTTC

AACGCTGGTGCGCCAGATAGAAGCGGGCGCGCCGGCGGATCTCTTCATCTCTGCTGATCAGAAATGGATGGATTACGCAG

CCGATAAAAAAGCGGTGGATACCACGACGCGCGAAACATTGCTTGGCAATAGCCTGGTGGTGGTAGCGCCGAAAGCCAGC

GAGCAGAAACCGTTTACCATCGACAACAAAACGGACTGGATTCGTCTGCTGAACGGCGGACGTCTGGCGGTGGGCGATCC

GCAACACGTACCGGCGGGAATTTATGCCAAAGAAGCGCTGCAAAAGTTGGGCGCATGGCAAACGCTTGAACCGAAACTGG

CGCCGGGCGAGGATGTGCGCGGCGCGTTGGCGCTGGTCGAACGTAACGAAGCGCCTTTGGGGCATTGTATACGGTTCTAA

TAGGCGGGGGGGGAAA

>16-Sbon-5

CCCTTAAAGGGTTCGGGTCAGAGACAGTCCAGACAGAACCGCTGATTTCCCCAAACGCCCGCTCAGTCGGGCGTTTTTGT

TACTCAAGATCCTGCTTTTCCTGCCGATATTTTCTGTGTTTAATCGTTAATGGGTATGAATAACCGCTGGGAAACCCCTG

AGATTATCGTTATATTGTTGTTTACATAACGAAACGCACAAGGAGTTACAGATGGCGCATTCCTGGTTACGCCTGGTCGC

AGGGGCGACATTATCTTTCGTCATCGCTGGTCATGCGCTGGCGGATGAAGGCAAGATCACCGTATTCGCCGCCGCGTCGC

TAACGAACGCGATGCAAGACATTGCGGCAGAATATAAAAAAGAGAAAAATGTGGATGTGGTCTCCTCATTCGCCTCCTCT

TCAACGCTGGCGCGCCAGATAGAAGCGGGCGCGCCGGCGGACCTGTTCATCTCTGCTGATCAGAAATGGATGGATTACGC

AGCCGATAAAAAAGCGGTGGATACCACGACGCGCGAAACATTGCTTGGCAATAGCCTGGTAGTGGTAGCGCCGAAAGCCA

GCGAGCAGAAACCGTTTACCATCGACAACAAAACGGACTGGATTCATCTGCTGAACGGCGGGCGTCTGGCGGTGGGCGAT

CCGCAACACGTACCGGCGGGAATTTATGCCAAAGAAGCGCTGCAAAAGCTGGGCGCATGGCAAACGCTTGAACCGAAACT

GGCGCCGGGCGAGGATGTGCGCGGTGCGTTGGCGTTGGTCGAACGTAACGAAGCGCCTTTAGGCATTGTATACGGTTCGA

AAGGCCGGGTGAG

>16-Sbon-6

CCAAATTAGGGTCGGTCAGAGACAGTCCAGACAGAACCGCTGATTTCCCCAAACGCCCGCTCAGTCGGGCGTTTTTGTTA

CTCAAGATCCTGCTTTTCCTGCCGATATTTTCTGTGTTTAATCGTTAATGGGTATGAATAACCGCTGGGAAACCCCTGAG

ATTATCGTTATATTATCGTTTACATAACGAAACGCACAAGGAGTTACAGATGGCGCATTCCTGGTTACGCCTGGTCGCAG

GGGCGACATTATCTTTCGTCATCGCTGGTCATGCGCTGGCGGATGAAGGCAAGATCACCGTATTTGCCGCCGCGTCGCTA

ACGAACGCGATGCAAGACATTGCGGCAGAATATAAAAAAGAGAAAAATGTGGATGTGGTCTCCTCATTCGCCTCCTCTTC

AACGCTGGCGCGCCAGATAGAAGCGGGCGCGCCGGCGGATCTGTTCATCTCTGCTGACCAGAAATGGATGGATTACGCAG

CCGATAAAAAAGCGGTGGATACCACGACGCGCGAAACATTGCTTGGCAATAGCCTGGTGGTGGTGGCGCCGAAAGCCAGC

GAGCAGAAACCGTTTACCATCGACAACAAAACGGACTGGATTCGTCTGCTGAACGGCGGGCGTCTGGCGGTGGGCGATCC

GCAACACGTACCGGCGGGAATTTATGCCAAAGAAGCGCTGCAAAAGTTGGGCGCATGGCAAACGCTTGAACCGAAACTGG

CGCCGGGCGAGGATGTGCGCGGCGCGTTGGCGCTGGTCGAACGTAACGAAGCGCCTTTGGGCATTGTATACGGTTCTAAA

TGGGCGGGTGAGA

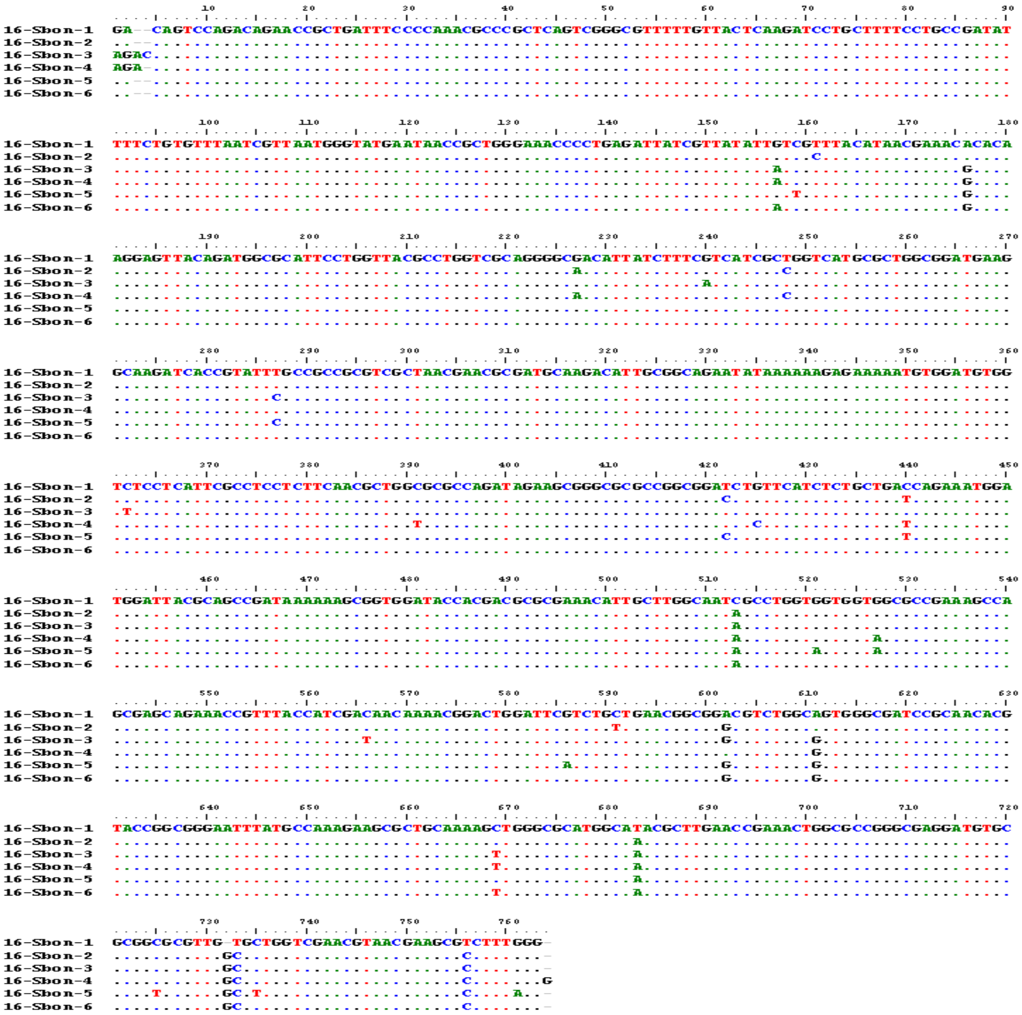

The designed SNP-based primers are based on the above align gene (*modA*) sequences (natural SNP is marked by red color and artificial mutated bases are marked by yellow shaded)

ModA-1-F: ACC CCT GAG ATT ATC GTT ATA CTG

ModA-1-R: ATC GCC CAC TGC CAG ATG T

ModA-2-F: CTG AGA TTA TCG TTA TAT TGT CGC

ModA-2-R: CCA GAC GCC CGC CGT TTA A

ModA-3-F: TCG CAG GGG CGA CAT TAT CTT CCA

ModA-3-R: AGA CGA ATC CAG TCC GTT TTG CTA

ModA-4-F: TTA CGC CTG GTC GCA GGG ACA

ModA-4-R: CAT TTC TGA TCA GCA GAG ATG GAG

ModA-5-F: CCT GAG ATT ATC GTT ATA TTA TT

ModA-5-R: ACG CCC GCC GTT CAG CAA AT

18-Sbon, Gene Name: Formimidoylglutamase (*hutG*)

**18-Sbon-F**	**GCATCTGGATCTGCGCCA**
**18-Sbon-R**	**TCGGCGACAAAGGTTCCC**

>18-Sbon-1

AAAGCGTAACCGAGCTAGCGTGTCGTCACTGCCGCAGCTATGCGACGCGCAGAGCCGCGCGTTACATTATGCCTGGTTCG

GCGTGAGCCGTGCGGCGAATACGCAGGCGTTGTGGCGGGAAGCGCAGTGGCGGAATGTTACCGTGGTGGAGGATCTGGAC

TGCCATGACGCGCTGGCGCAATATGACGCACATTATCGACAAGGTGGATAAAATTTATCTGACTATCGATCTCGACGTAT

TGCCTGTCTGGGAAATGCCGGCCGTCTCCGCTCCCGCAGCGCTGGGCGTGCCGCTGATACAGGTTCTGCGTTTAATTGAG

CCGGTTTGCCGCAGCGGAAAATTACAGGCGGCGGATCTGGTTGAATTTAATCCACGCTTTGATGAAGATGGCGCAGCGGC

GCGCGTGGCGGCGCGGCTTGGCTGGCAAATCGCGCACTGGTGGCGTTAATCTGTCATCCGGAAAACATTATCCGCATAAG

GAAGGCTTCACGCATGTATTCATCCCGCTCCCGTTCTGCACCTGCGCCTTTTTACGAAACGGTGAAACAGGACATCTGTA

AAAAAATAGCCGGCGGCGTCTGGCAGCCGCACGATCGCATTCCGTCGGAAGCAGAACTGGTCGCCCAGTATGGCTTTAGC

CGAATGACCATCAATCGGGCGCTGCGTGAGCTGACGGATGAAGGGTGGCTGGTGCGTTTACAGGGCGTGGGAACTTTTTT

CCCCCGA

>18-Sbon-2

ATGCCCATCGAGCTAGCGACGTCGTCACTGCGCGCACTCTATGCGACGCGCAGAGCGCGCGGCGTTACTTTATGCCTGGT

TCGGCGCGAGCCGTGCGGCGAATACGCAGGCGTTGTGGCGGGAAGCGCAGTGGCGGAATGTTACCGTGGTGGAGGATCTT

GACTGCCATGACGCGCTGGCGCAAATGACATGCATTATCGACAAGGTGGATAAAATTTATCTGACTATCGATCTCGACGT

ATTGCCTGTCTGGGAAATGCCGGCCGTCTCCGCTCCCGCAGCGCTGGGCGTGCCGCTGATACAGGTTCTGCGTTTAATTG

AGCCGGTTTGCCGCAGCGGAAAATTACAGGCGGCGGATCTGGTTGAGTTTAATCCACGCTTTGATGAAGATGGCGCAGCG

GCGCGCGTGGCGGCGCGGCTTGGCTGGCAAATCGCGCACTGGTGGCGTTAATCTGTCATCCGGAAAACACTCTCCGCATA

AGGAAGGCTTCACGCATGTATTCATCCCGCTCCCGTTCTGCACCTGCGCCTTTTTACGAAACGGTGAAACAGGACATCTG

TAAAAAAATAGCCGGCGGCGTCTGGCAGCCGCACGATCGCATTCCGTCGGAAGCTGAACTGGTCGCTCAGTATGGCTTTA

GCCGAATGACCATCAACCGGGCGCTGCGTGAGCTGACGGATGAAGGGTGGCTGGTGCGTTTACAGGGCGTGGGAACCTTT

GGTCGCCGAA

>18-Sbon-3

GTGCCCATCGAGCTAGCGTTTCGTCACTGGCGCAGCTATGCGACGCGCAGAGCCGCGCGTTTCATTATGCCTGTTTCGGC

GTGAGCCGTGCGGCGAATACGCAGGCGTTGTGGCGGGAAGCGCAGTGGCGGAATGTTACCGTGGTGGAGGATCTGGACTG

CCATGACGCGCTGGCGCAAATGGCGCAATTATCGACAAGGTGGATAAAATTTATCTGACTATCGATCTCGACGTATTGCC

TGTCTGGGAAATGCCGGCCGTCTCCGCTCCCGCAGCGCTGGGCGTGCCGCTGATACAGGTTCTGCGTTTAATTGAGCCGG

TTTGCCGCAGCGGAAAATTACAGGCGGCGGATCTGGTTGAGTTTAATCCACGCTTTGATGACGATGGCGCAGCGGCGCGC

GTGGCGGCGCGGCTTGGCTGGCAAATCGCGCACTGGTGGCGTTAATCTGTCATCCGGAAAACACTATCCGCATAAGGAAG

GCTTCACGCATGTATTCATCCCGCTCCCGTTCTGCGCCTGCGCCTTTTTACGAAACGGTGAAACAGGACATCTGTAAAAA

AATAGCCGGCGGCGTCTGGCAGCCGCACGATCGCATTCCGTCGGAAGCTGAACTGGTCGCCCAGTATGGCTTTAGCCGAA

TGACCATCAACCGGGCGCTGCGTGAGCTGACGGATGAAGGGTGGCTGGTGCGTTTACAGGGCGTGGGAACTTT

>18-Sbon-4

TGCCCATTCGAGCTAGCGTTTCGTCACTGGCGCAGTCTATGCGACGCGCAGAGCCGCGCGTTTCATTATGCCTGTTTCGG

CGTGAGCCGTGCGGCGAATACGCAGGCGTTGTGGCGGGAAGCGCAGTGGCGGAATGTTACCGTGGTGGAGGATCTGGACT

GCCATGACGCGCTGGCGCAAATGGCGCAATTATCGACAAGGTGGATAAAATTTATCTGACTATCGATCTCGACGTATTGC

CTGTCTGGGAAATGCCGGCCGTCTCCGCTCCCGCAGCGCTGGGCGTGCCGCTGATACAGGTTCTGCGTTTAATTGAGCCG

GTTTGCCGCAGCGGAAAATTACAGGCGGCGGATCTGGTTGAGTTTAATCCACGCTTTGATGAAGATGGCGCAGCGGCGCG

CGTGGCGGCGCGGCTTGGCTGGCAAATCGCGCACTGGTGGCGTTAATCTGTCATCCGGAAAACATTATCCGCATAAGGAA

GGCTTCACGCATGTATTCATCCCGCTCCCGTTCTGCACCTGCGCCTTTTTACGAAACGGTGAAACAGGACATCTGTAAAA

AAATAGCCGGCGGCGTCTGGCAGCCGCACGATCGCATTCCGTCGGAAGCAGAACTGGTCGCCCAGTATGGCTTTAGCCGA

ATGACCATCAATCGGGCGCTGCGTGAGCTGACGGATGAAGGGTGGCTGGTGCGTTTACAGGGCGTGGGAACTTTGGGTCG

CCGAAA

>18-Sbon-5

GTGGCTCCTCCGAGCTAGCGACGTCGTCACTGGCGCAGCTATGCGACGCGCAGAGCCGCGGGTTACATTATGCTTGGTTC

GGCGTGAGCCGTGCGGCGAATACGCAGGCGTTGTGGCGGGAAGCGCAGTGGCGGAATGTTACCGTGGTGGAGGATCTGGA

CTGCCATGACGCGCTGGCGCATATGACGCCATTATTGACAAGGTGGATAAAATTTATCTGACTATCGATCTCGACGTATT

GCCTGTCTGGGAAATGCCGGCCGTCTCCGCTCCCGCAGCGCTGGGCGTGCCGCTGATACAGGTTCTGCGTTTAATTGAGC

CGGTTTGCCGCAGCGGAAAATTACAGGCGGCGGATCTGGTTGAATTTAATCCACGCTTTGATGAAGATGGCGCAGCGGCG

CGCGTGGCGGCGCGGCTTGGCTGGCAAATCGCGCACTGGTGGCGTTAATCTGTCATCCGGAAAACATTATCCGCATAAGG

AAGGCTTCACGCATGTATTCATCCCGCTCCCGTTCTGCGCCTGCGCCTTTTTACGAAACGGTGAAACAGGACATCTGTAA

AAAAATAGCCGGCGGCGTCTGGCAGCCGCACGATCGCATTCCGTCGGAAGCTGAACTGGTCGCTCAGTATGGCTTTAGCC

GAATGACCATCAACCGGGCGCTGCGTGAGCTGACGGATGAAGGGTGGCTGGTGCGTTTACAGGGCGTGGGAACCTTTGGT

CGCCGAA

>18-Sbon-6

AAGCCATCGACTAGCCGTCTCGTCACTGCGCGCAGCTATGCTGACGCGCAGAGCCGCGCGTTTCATTATGCCTGTTTCGG

CGTGAGCCGTGCGGCGAATACGCAGGCGTTGTGGCGGGAAGCGCAGTGGCGGAATGTTACCGTGGTGGAAGATCTGGACT

GCCGTGACGCGCTGGCGCAAATGACACACATTATCGACAAGGTGGATAAAATTTATCTGACTATCGATCTCGACGTATTG

CCTGTCTGGGAAATGCCGGCCGTCTCCGCTCCCGCAGCGCTGGGCGTGCCGCTGATACAGGTTCTGCGTTTAATTGAGCC

GGTTTGCCGCAGCGGAAAATTACAGGCGGCGGATCTGGTTGAGTTTAATCCACGCTTTGATGACGATGGCGCAGCGGCGC

GCGTGGCGGCGCGGCTTGGCTGGCAAATCGCGCACTGGTGGCGTTAATCTGTCATCCGGAAAACACTATCCGCATAAGGA

AGGCTTTACGCATGTATTCATCCCGCTCCCGTTCTGCGCCTGCGCCTTTTTACGAAACGGTGAAACAGGACATCTGTAAA

AAAATAGCCGGCGGCGTCTGGCAGCCGCACGATCGCATTCCGTCGGAAGCAGAACTGGTCGCCCAGTATGGCTTTAGCCG

AATGACCATCAACCGGGCGCTGCGTGAGCTGACGGATGAAGGGTGGCTGGTGCGTTTACAGGGCGTGGGAACTTTTGTCG

CCCGAA

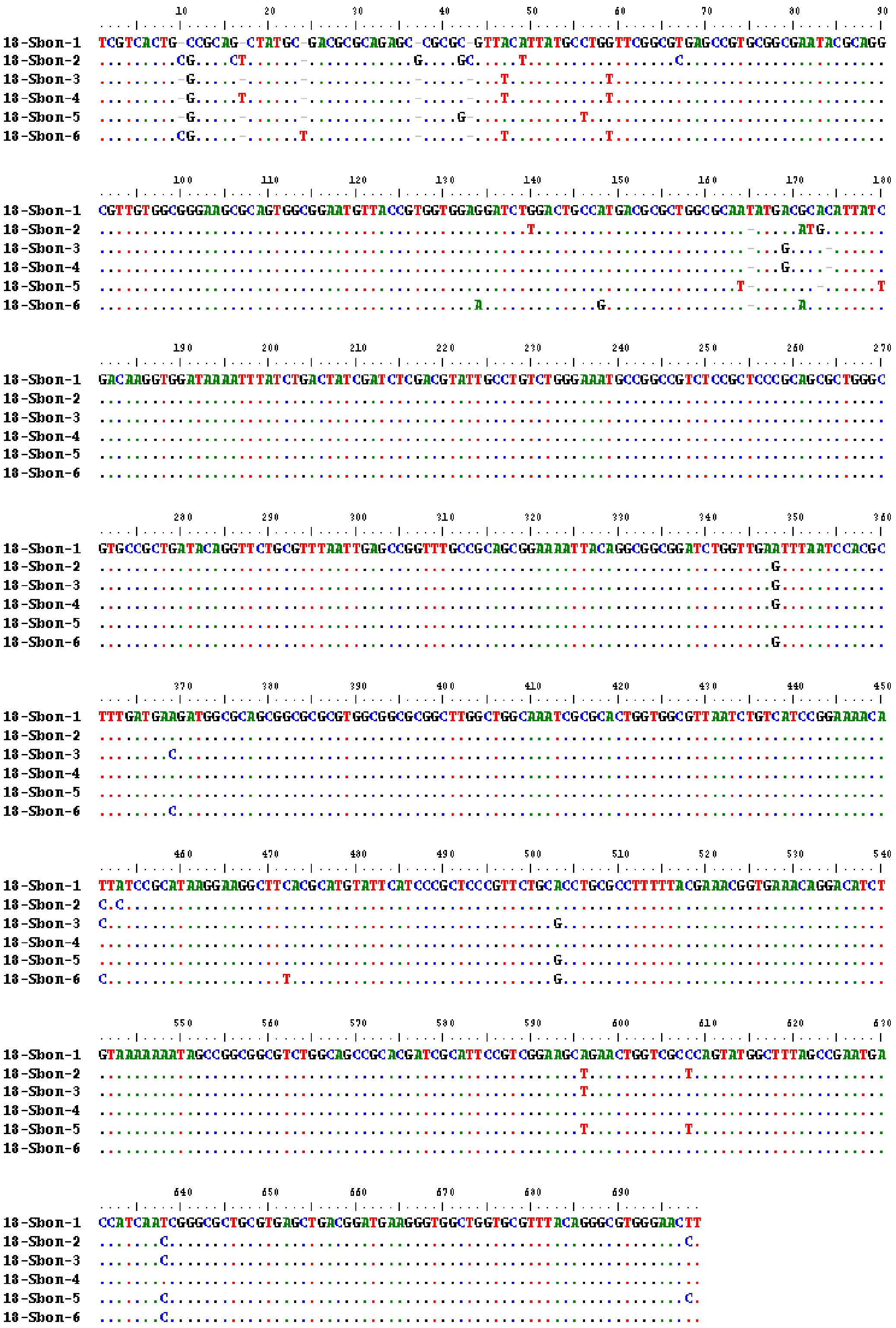

The above alignment of gene, formimidoylglutamase (hutG) did not produce the all target sequences of Salmonella, where we did not find any suitable SNPs.

19-Sbon, Gene Name: Hypothetical ABC transporter ATP-binding (*yehX*)

**19-Sbon-F**	**TCACGGCGGGTAAGAGGA**
**19-Sbon-R**	**ATGAGATTCGCCAGGCCG**

>19-Sbon-1

CGCGGGCTGGTTCTCATGATAGATATCCGGTTCGTGCATCAGCGAGCAGGCCAGCGCCAGTCGCTGTTTAAAGCCCAGCG

GCAGCGCATCGGTGGGATGCGACGCGATGCTTTTCAGACCAAACGCTTCACTCATGCGCTGAATTTTTTCATTTTGCGCG

CGGCCCCGCAAACCGTAGACGCCGGAGAAAAAACGCAGGTTCTGCTCAACCGTCAGGTTGCCGTAAAGCGAAAATTTTTG

CGCCATATAGCCCAGATGCTGACGGGCTTTGCCGGAGCTGACTTTCAGGTCCATATCCAGCACCAGCGCCTTGCCGGACG

TCGGCACCAGCAGGCCGCACATCATTTTAAAGGTGGTTGATTTACCCGCGCCGTTCGGGCCGAGCAGGCCAAAAATCTCG

CCGCGCTGTACGACGAAATTAACATGGTCCGTAGCCGCGAAATCGCCAAATTTTTTCGTCAGTTCCTGCGCTTCAATCAC

CGTTTCGCCGGCAGTGCCTTCAACCGTATGCAGGATTGAACCCAGCGGAGACTCCGAGGTTCCCGCTCCGCCAAGCAGAT

CGATAAACGCATCTTCGAAGCGCGGCGCGGTCTCATTGAGGGTGATTTCCGGCATCCCTTCGGCCTGGAAAAATTCTCAT

A

>19-Sbon-2

GTCCGGGCATGGTTCTCAAGGCATATATATCCGGTTCGTGCATCAGCGAGCAGGCCAGCGCCAGTCGCTGTTTAAAGCCC

AGCGGCAGCGCATCGGTGGGATGCGACGCGATGCTTTTCAGACCAAACGCTTCACTCATGCGCTGAATTTTTTCATTTTG

CGCGCGGCCCCGCAAACCGTAGACGCCGGAGAAAAAACGCAGGTTCTGCTCAACCGTCAGGTTGCCGTAAAGCGAAAATT

TTTGCGCCATATAGCCCAGATGCTGACGGGCTTTGCCGGAGCTGACTTTCAGGTCCATATCCAGCACCAGCGCCTTGCCG

GACGTCGGCACCAGCAGGCCGCACATCATTTTAAAGGTGGTTGATTTACCCGCGCCGTTCGGGCCGAGCAGGCCAAAAAT

CTCGCCGCGTTGTACGACGAAATTAACATGGTCCGTGGCCGCGAAATCGCCAAATTTTTTCGTCAGTTCCTGCGCTTCAA

TCACCGTTTCGCCGGCAGTGCCTTCAACCGTATGCAGGATTGAACCCAGCGGAGACTCCGAGGTTCCCGCTCCGCCAAGC

AGATCGATAAACGCATCTTCGAAGCGCGGCGCGGTCTCATTGAGGGTGATTTCCGGCATTCCTTCGGCCTGCGAAATCTC

AT

>19-Sbon-4

GAGGGGCATGGTCTCATGATATATATCCGGTTCGTGCATCAGCGAGCAGGCCAGCGCCAGTCGCTGTTTAAAGCCCAGCG

GCAGCGCATCGGTGGGATGCGACGCGATGCTTTTCAGACCAAACGCTTCACTCATGCGCTGAATTTTTTCATTTTGCGCG

CGGCCCCGCAAACCGTAGACGCCGGAGAAAAAACGCAGGTTCTGCTCAACCGTCAGGTTGCCGTAAAGCGAAAATTTTTG

CGCCATATAGCCCAGATGCTGACGGGCTTTGCCGGAGCTGACTTTCAGGTCCATATCCAGCACCAGCGCCTTGCCGGACG

TCGGCACCAGCAGGCCGCACATCATTTTAAAGGTGGTTGATTTACCCGCGCCGTTCGGGCCGAGCAGGCCAAAAATCTCG

CCGCGCTGTACGACGAAATTAACATGGTCCGTAGCCGCGAAATCGCCAAATTTTTTCGTCAGTTCCTGCGCTTCAATCAC

CGTTTCGCCGGCAGTGCCTTCAACCGTATGCAGGATTGAACCCAGCGGAGACTCCGAGGTTCCCGCTCCGCCAAGCAGAT

CGATAAACGCATCTTCGAAGCGCGGCGCGGTCTCATTGAGGGTGATTTCCGGCATCCCTTCGGCCTGCGAAATCTCAT

>19-Sbon-5

GTGGGGCATGGTTATCACGATACATATCCGGTTCGTGCATCAGCGAGCAGGCCAGCGCCAGTCGCTGTTTAAAACCCAGC

GGCAGCGCATCGGTGGGATGCGACGCGATGCTTTTCAGACCAAATGCTTCACTCATGCGCTGAATTTTTTCATTTTGCGC

GCGGCCCCGCAAACCGTAGACGCCGGAGAAAAAACGCAGGTTCTGCTCAACCGTCAGGTTGCCGTAAAGCGAAAATTTTT

GCGCCATATAGCCCAGATGCTGACGGGCTTTGCCGGAGCTGACTTTCAGGTCCATATCCAGCACCAGCGCCTTGCCGGAC

GTCGGCACCAGCAGGCCGCACATCATTTTAAAGGTGGTTGATTTACCCGCGCCGTTCGGGCCGAGCAGGCCAAAAATCTC

GCCGCGTTGTACGACGAAATTAACATGGTCCGTGGCCGCGAAATCGCCAAATTTTTTCGTCAGTTCCTGCGCTTCAATCA

CCGTTTCGCCGGCAGTGCCTTCAACCGTATGCAGGATTGAACCCAGCGGAGACTCCGAGGTTCCCGCTCCGCCAAGCAGA

TCGATAAACGCATCTTCGAAACGCGGCGCGGTCTCATTGAGGGTGATTTCCGGCATCCCTTCGGCCTGGCGAATCTAATG

A

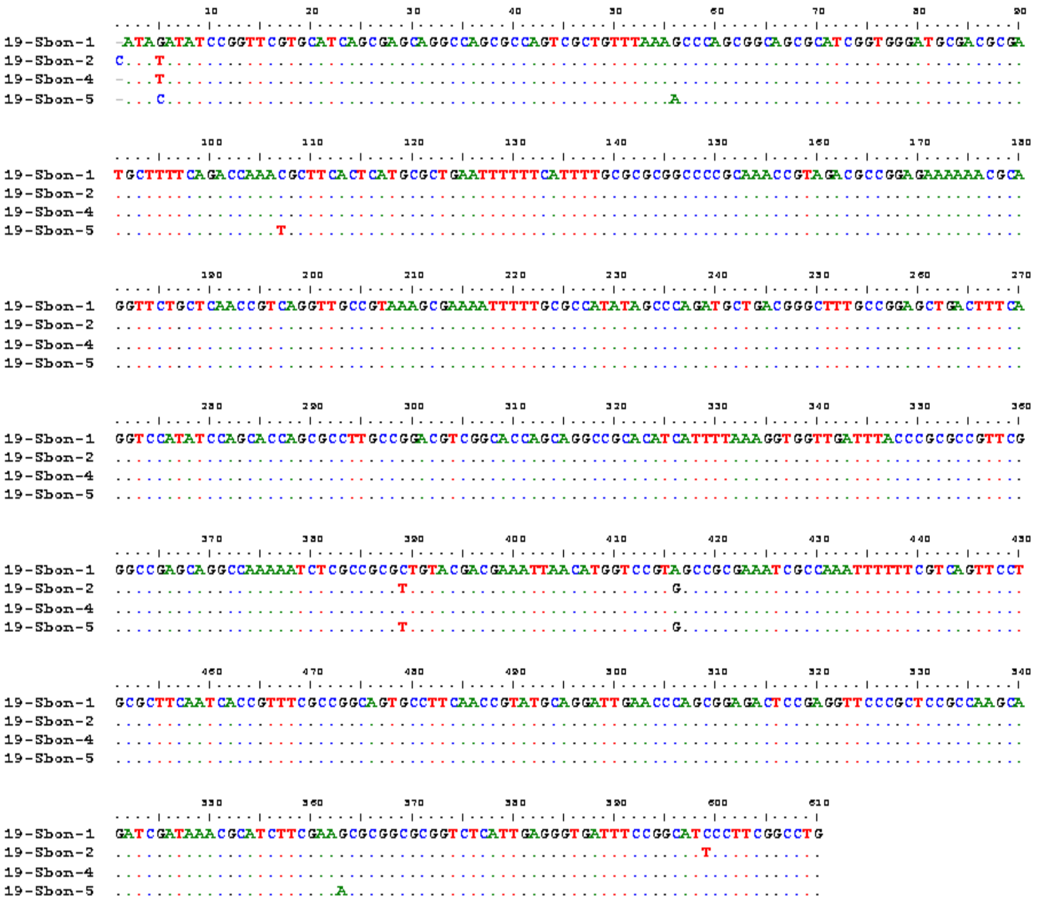

The above alignment of the gene, hypothetical ABC transporter ATP-binding (yehX) did not produce the all target sequences of Salmonella, where we did not find any suitable SNPs.

21-Sbon, Gene Name: pyruvate formate-lyase 3-activating enzyme (*ybiY*)

**16-Sbon-F**	**CTGCTTAAACGGCGCGTC**
**16-Sbon-R**	**TGGTGCGGCATGATCCTG**

>21-Sbon-3

CGGGGGTTGCTGTCTGCAGATGTATGGCGGTCTATAACCGGGATGTAGTGAAAGTACCTAAAGGCTACCACCCGAGTTGC

CACCATCGCGGGTTACGATAACTACTATCTGAACGTGATGGCCGGCCCGCTACGAAAATGGCGTTTCACCTGGGAAGAGA

ACCACGCGTGGATTAACTCACCAGACTACCCGCGATAACACTTCGTTGAGTGGTACTTTGCCAGCCGTCCTCTGCGGCTG

GCTTTTTTATTCAACTCTGCGCCACACACGCTCATAAGATCGCTATCCGCCTTCTCTTAAATTATTTCCCTGTTTTGTAT

ATAGCGTATTTACGGCACGGCCTCCACCAGGAATACGCAATAAAATAATGATGCAGAAGAAGACTTACCCTATTTGCCGA

TATTATTATGGATCAAGTAGTTACGTAGAGGTGGTAAACACTGGGGAGGTTTCAACAGGGTATATCCTGTGCGCACTCGT

GGAGGGAGTAAAGGTTGTGTAACCAGCGGAGAATATCTCTGGATATTCTCCGCTAATATTGGAGAGGTATCAGGCTATTA

CATCGCGACGTTTCCCCTGAATTTCACGCAGCTTGTTCTCGGCCAGCCGTTCAATTTCTGTTAGCGGCACCCCTTTTGTT

TCCGGCACATAACGAGAAATAAAGACATAGCAGACCAATTAAAGACGACAAAAATCCACATCGAGAAAGCGCCGCCGAAG

GTCTCCTGCAGCCAGGCGTTATCATTTATGACCGGGAACAACAGTGAGATGAGGAAGTTGGCTATCCACATCAGGCTCAC

CGCCCAGCCCCATTCCCAAAACCTTTTTATCTTTTTTCAGGGAAAACCTTCAGAATCAGTACCCATGCG

>21-Sbon-4

TTGGCAGCATCTGCAGATGTATGGCGGTCTATAACCGGGATGTAGTGAAAGTACCTAAGGGCTACCACCCGGTTGCCACC

ATCGCGGGTTACGATAACTACTATCTGAACGTGATGGCCGGCCCGCTACGAAAATGGCGTTTCACCTGGGAAGAGAACCA

CGCGTGGATTAACTCACCAGACTACCCGCGATAACACTTCGTTGAGTGGTACTTTGCCAGCCGTCCTCTGCGGCTGGCTT

TTTTATTCAACTCTGCGCCACACACGCTCATAAGATCGCTATCCGCCTTCTCTTAAATTATTTCCCTGTTTTGTATATAG

CGTATTTACGGCACGGCCCCCACCAGGAATACGCAATAAAATAATGATGCAGAAGAAGACTTACCCTATTTGCCGATATT

ATTATGGATCAATTAGTTACGTAGAGGTGGTAAACACTGGGGAGGTTTCAACAGGGTATATCCTGTGCGCACTCGCTGGA

GGGAGTAAAGGTTGTGTAACCAGCGGAGAATATCTCTGGATATTCTCCGCTAATATTGGAGAGGTATCAGGCTATTACAT

CGCGACGTTTCCCCTGAATTTCACGCAGCTTGTTCTCGGCCAGCCGTTCAATTTCTGTTAGCGGCACCCCTTTTGTTTCC

GGCACATAACGAGAAATAAAGACATAGCAGACCAAATTAAAGACGACAAAAATCCACATCGAGAAAGCGCCGCCGAAGGT

CTCCTGCAGCCAGGCGTTATCATTTATGACCGGGAACAACAGTGAGATGAGGAAGTTGGCTATCCACATCAGACTCACCG

CCAGCCCCATCCCAAAACCTTTTATCTTTTCAGGGGAAAACCTCAGAAATCAGTACCCATGCGGCCAACGCTCCAGCTGA

CGGCAAACAGCAGCATAAAAAAACAGGGATGCCGAAGATGGGTATTGTAGCCTGTATCGTGGGTCGTACAAAGCCGTAAT

GACGCGATCAACAGGCCAGAGATTGTATCCGATGGTACCGAGTTTTCATAAATGCGGATATAGCGGTCATCAGCGGTGGA

AGATGGGAATCAGTGTCCGACGCCCACAATCTATACAATAGGGAGATAA

```
                   10        20        30        40        50        60        70        80        90
          ....|....|....|....|....|....|....|....|....|....|....|....|....|....|....|....|....|....|
21-Sbon-3 CGGGGGTTGCTGTCTGCAGATGTATGGCGGTCTATAACCGGGATGTAGTGAAAGTACCTAAAGGCTACCACCCGAGTTGCCACCATCGCG
21-Sbon-4 --TT..CA..-A.................................................G............-...............

                  100       110       120       130       140       150       160       170       180
          ....|....|....|....|....|....|....|....|....|....|....|....|....|....|....|....|....|....|
21-Sbon-3 GGTTACGATAACTACTATCTGAACGTGATGGCCGGCCCGCTACGAAAATGGCGTTTCACCTGGGAAGAGAACCACGCGTGGATTAACTCA
21-Sbon-4 ..........................................................................................

                  190       200       210       220       230       240       250       260       270
          ....|....|....|....|....|....|....|....|....|....|....|....|....|....|....|....|....|....|
21-Sbon-3 CCAGACTACCCGCGATAACACTTCGTTGAGTGGTACTTTGCCAGCCGTCCTCTGCGGCTGGCTTTTTTATTCAACTCTGCGCCACACACG
21-Sbon-4 ..........................................................................................

                  280       290       300       310       320       330       340       350       360
          ....|....|....|....|....|....|....|....|....|....|....|....|....|....|....|....|....|....|
21-Sbon-3 CTCATAAGATCGCTATCCGCCTTCTCTTAAATTATTTCCCTGTTTTGTATATAGCGTATTTACGGCACGGCCTCCACCAGGAATACGCAA
21-Sbon-4 ........................................................................C.................

                  370       380       390       400       410       420       430       440       450
          ....|....|....|....|....|....|....|....|....|....|....|....|....|....|....|....|....|....|
21-Sbon-3 TAAAATAATGATGCAGAAGAAGACTTACCCTATTTGCCGATATTATTATGGATCAAGTAGTTACGTAGAGGTGGTAAACACTGGGGAGGT
21-Sbon-4 ........................................................T.................................

                  460       470       480       490       500       510       520       530       540
          ....|....|....|....|....|....|....|....|....|....|....|....|....|....|....|....|....|....|
21-Sbon-3 TTCAACAGGGTATATCCTGTGCGCACTCG-TGGAGGGAGTAAAGGTTGTGTAACCAGCGGAGAATATCTCTGGATATTCTCCGCTAATAT
21-Sbon-4 .............................C............................................................

                  550       560       570       580       590       600       610       620       630
          ....|....|....|....|....|....|....|....|....|....|....|....|....|....|....|....|....|....|
21-Sbon-3 TGGAGAGGTATCAGGCTATTACATCGCGACGTTTCCCCTGAATTTCACGCAGCTTGTTCTCGGCCAGCCGTTCAATTTCTGTTAGCGGCA
21-Sbon-4 ..........................................................................................

                  640       650       660       670       680       690       700       710       720
          ....|....|....|....|....|....|....|....|....|....|....|....|....|....|....|....|....|....|
21-Sbon-3 CCCCTTTTGTTTCCGGCACATAACGAGAAATAAAGACATAGCAGACCAA-TTAAAGACGACAAAAATCCACATCGAGAAAGCGCCGCCGA
21-Sbon-4 .................................................A........................................

                  730       740       750       760       770       780       790       800       810
          ....|....|....|....|....|....|....|....|....|....|....|....|....|....|....|....|....|....|
21-Sbon-3 AGGTCTCCTGCAGCCAGGCGTTATCATTTATGACCGGGAACAACAGTGAGATGAGGAAGTTGGCTATCCACATCAGGCTCACCGCCCAGC
21-Sbon-4 ............................................................................A.........-...

                  820       830       840       850       860       870       880       890       900
          ....|....|....|....|....|....|....|....|....|....|....|....|....|....|....|....|....|....|
21-Sbon-3 CCCATTCCCAAAACCTTTTTATCTTTTTTCAGGGAAAACCTTCAGAATCAGTACCCATGCG-----------------------------
21-Sbon-4 .....-.............-.......CA-G..........CAGA................GCCAACGCTCCAGCTGACGGCAAACAGCA

                  910       920       930       940       950       960       970       980       990
          ....|....|....|....|....|....|....|....|....|....|....|....|....|....|....|....|....|....|
21-Sbon-3 ------------------------------------------------------------------------------------------
21-Sbon-4 GCATAAAAAAACAGGGATGCCGAAGATGGGTATTGTAGCCTGTATCGTGGGTCGTACAAAGCCGTAATGACGCGATCAACAGGCCAGAGA

                 1000      1010      1020      1030      1040
          ....|....|....|....|....|....|....|....|....|....|....|...
21-Sbon-3 ----------------------------------------------------------
21-Sbon-4 TTGTATCCGATGGTACCGAGTTTTCATAAATGCGGATATAGCGGTCATCAGCGGTGGA
```

The above alignment of the gene, pyruvate formate-lyase 3-activating enzyme (ybiY)did not produce the all target sequences of Salmonella, where we did not find any suitable SNPs.
